# Physiological Appetite Regulation and Bariatric Surgery

**DOI:** 10.3390/jcm13051347

**Published:** 2024-02-27

**Authors:** Indra Ramasamy

**Affiliations:** Department of Blood Sciences, Conquest Hospital, Hastings TN37 7RD, UK; indrar@ozemail.com.au

**Keywords:** hunger centre, satiety centre, POMC, AgRP/NPY, leptin, incretin, bariatric surgery, obesity therapy, obesity genetics

## Abstract

Obesity remains a common metabolic disorder and a threat to health as it is associated with numerous complications. Lifestyle modifications and caloric restriction can achieve limited weight loss. Bariatric surgery is an effective way of achieving substantial weight loss as well as glycemic control secondary to weight-related type 2 diabetes mellitus. It has been suggested that an anorexigenic gut hormone response following bariatric surgery contributes to weight loss. Understanding the changes in gut hormones and their contribution to weight loss physiology can lead to new therapeutic treatments for weight loss. Two distinct types of neurons in the arcuate hypothalamic nuclei control food intake: proopiomelanocortin neurons activated by the anorexigenic (satiety) hormones and neurons activated by the orexigenic peptides that release neuropeptide Y and agouti-related peptide (hunger centre). The arcuate nucleus of the hypothalamus integrates hormonal inputs from the gut and adipose tissue (the anorexigenic hormones cholecystokinin, polypeptide YY, glucagon-like peptide-1, oxyntomodulin, leptin, and others) and orexigeneic peptides (ghrelin). Replicating the endocrine response to bariatric surgery through pharmacological mimicry holds promise for medical treatment. Obesity has genetic and environmental factors. New advances in genetic testing have identified both monogenic and polygenic obesity-related genes. Understanding the function of genes contributing to obesity will increase insights into the biology of obesity. This review includes the physiology of appetite control, the influence of genetics on obesity, and the changes that occur following bariatric surgery. This has the potential to lead to the development of more subtle, individualised, treatments for obesity.

## 1. Introduction

The gut endocrine system and the gut-central nervous system axis function to digest and absorb food and, consequently, to regulate appetite. The gut-pancreatic endocrine system is one of the largest endocrine systems in the body. Most of the gut hormones secreted (incretins, cholecystokinin, and pancreatic polypeptide), except ghrelin, act to increase satiety and decrease food intake [1]. The adipose tissue is widely distributed in the body; has distinct functions both in energy homeostasis and as an endocrine organ. Leptin, an adipokine secreted by the adipose tissue, has a satiety effect on the central nervous system [2]. Many gut hormones act on the hypothalamus and brain stem areas of appetite control. Within the arcuate nucleus of the hypothalamus are the proopiomelanocortin (POMC)-secreting neurons, which suppress appetite, and a second population of neurons, which increase food intake and co-express neuropeptide Y (NPY) and agouti-related protein (AgRP) [3].

Obesity has become a major contributor to the global burden of chronic disease. Obesity is associated with several disease states, which include, among others, type 2 diabetes (T2DM), hypertension, dyslipidemia, cardiovascular disease, non-alcoholic fatty liver disease, and obstructive sleep apnea [4]. This has led to the development of several obesity treatment methods, including bariatric surgery. 

Appropriate response to changes in the homeostatic control of energy intake is obligatory for a stable energetic state and weight maintenance. Understanding the mechanisms controlling appetite and weight transformation may facilitate the understanding of appetite changes as well as weight loss and hormonal changes following bariatric surgery. Bariatric surgery is associated with side effects such as dumping syndrome, postprandial hypoglycaemia, partial T2DM remission or T2DM relapse, and weight regain. Knowledge of the mechanisms of appetite homeostasis may facilitate a greater understanding of changes following bariatric surgery, the development of therapeutic approaches for avoiding the side effects of bariatric surgery, and the advancement of less invasive weight loss strategies [5].

The main goal of this review is to summarise recent advances in the physiology of energy homeostasis, the genetics of obesity and appetite regulation, and the changes that arise following bariatric surgery. The analyses will have clinical relevance in identifying future developments in treatment for mitigating the risk of obesity and its comorbidities. 

## 2. Method

This narrative review covered several topics, and individual terms, e.g., incretins and leptin, were used to identify reviews or articles in PubMed, MEDLINE, Google Scholar, and Web of Science over the past 15 years. From each article/review we extracted further references for the studies included in this review.

## 3. Neurons and the Control of Appetite

**Figure 1:**
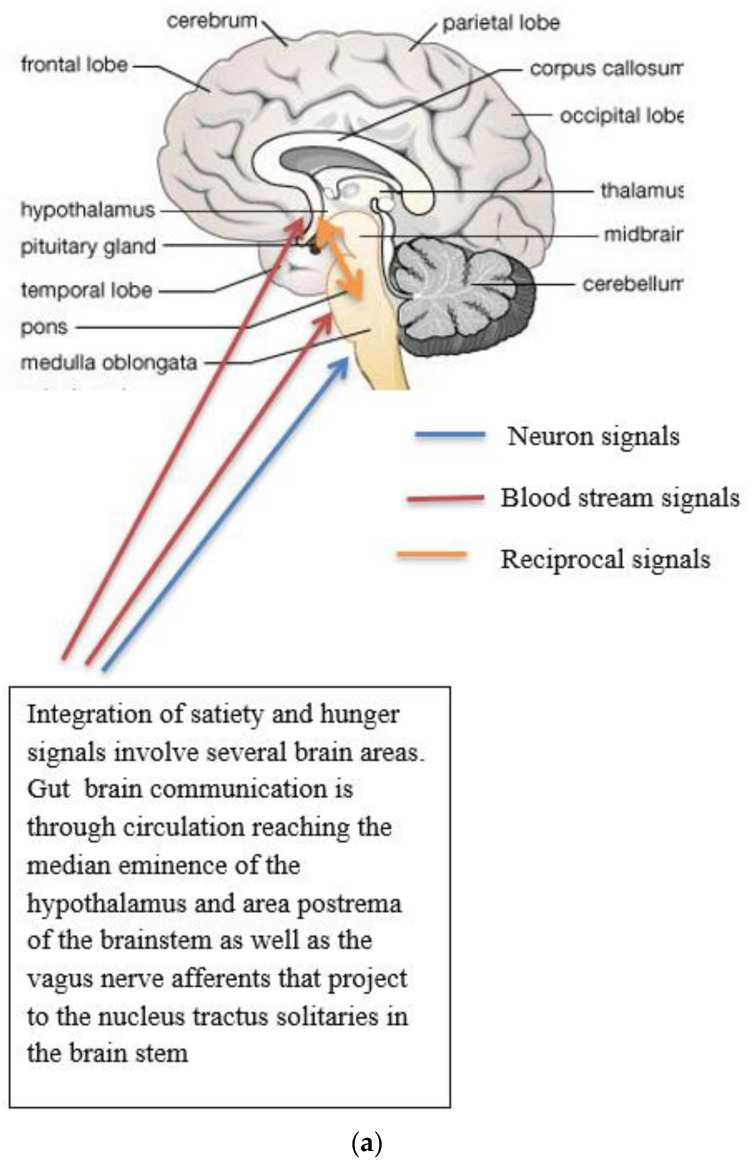

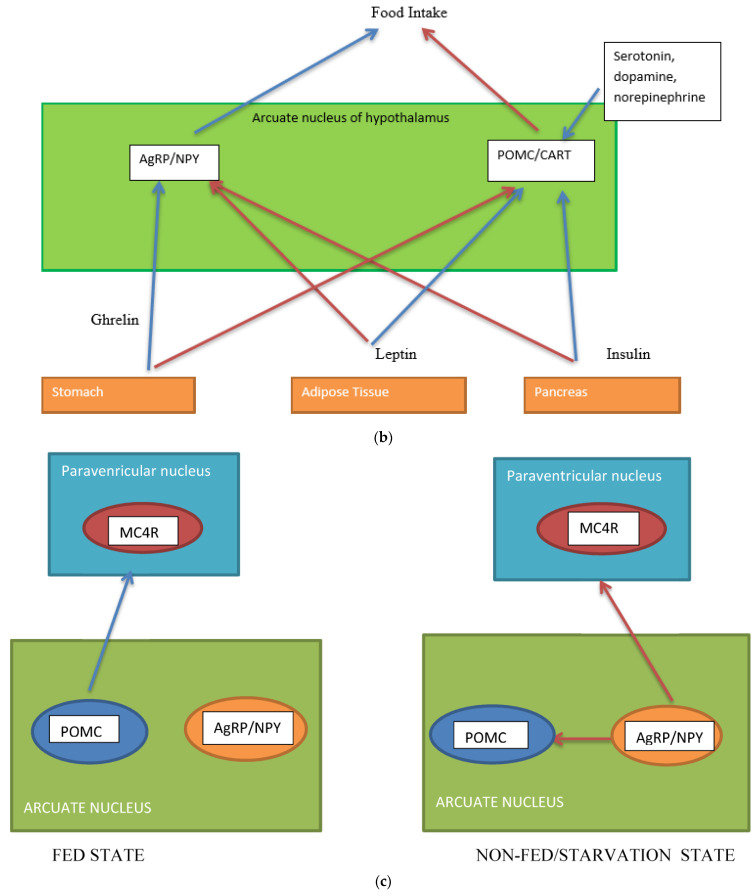
(**a**) Gut-brain communication underlying appetite control; (**b**) neuron function in appetite control, AgRP/NPY (neuropeptide Y/agouti-related peptide neurons), POMC (proopiomelanocortin), 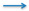 activation, 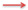 inhibition; (**c**) neuron interaction in appetite control, activation 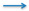, inhibition 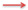. Populations of POMC neurons express different receptors. e.g., leptin and insulin. In the fed state, POMC neurons act to decrease food intake and increase energy expenditure. In the fasted state, AgRP/NPY neurons inhibit the activity of MC4R neurons and POMC neurons.

Several neuronal populations, which are distributed throughout the brain, affect the capacity for food intake. Specific areas of the hypothalamus are believed to control feeding behaviour. Two distinct neuronal populations are thought to be significant in regulating energy balance. These are found in the arcuate nucleus of the hypothalamus (ARH), the anorexigenic (appetite suppressing) proopiomelanocortin (POMC) neurons, and the orexigenic (appetite-increasing) neuropeptide Y (NPY)/agouti-related peptide (AgRP) co-expressing neurons. The neurons are positioned to receive signals from both peripheral organs due to the rich blood supply to the arcuate nucleus as well as input from various parts of the central nervous system [6]. POMC-expressing neurons are also located in the nucleus tractus solitaries (NTS) and have different behavioral energy homeostasis functions in the ARH and NTS [7]. POMC neurons have been investigated extensively because of their central role in energy balance. POMC neurones in the ARH extend fibres to other ARH neurones and other multiple brain regions, including the paraventricular nucleus of the hypothalamus, lateral hypothalamus, commissural nucleus of the solitary tract, bed nucleus of the stria terminalis, nucleus accumbens, septal nucleus, ventral tegmental area, central amygdala, periaqueductal grey, and dorsal raphe nucleus [8]. The ARH contains distinct populations of POMC neurons that are either GABAergic or glutamatergic neurotransmitters and are activated by serotonin 5-HT2_C_s, insulin, and leptin receptors (among others) [8]. The evidence for leptin acting on a different population of POMC neurons than insulin is conflicting [9]. Other studies suggest a heterogeneous population for POMC and that POMC neurons form several molecularly distinct clusters [10]. POMC and AgRP neurons change neuronal activity in response to glucose fluctuations [11]. Specific responses may be segregated into distinct populations of POMC neurons (Figure 1a–c). POMC neurons suppress appetite, and following processing of POMC by proprotein convertase (coded by *PCSK1*), release α-melanocyte stimulating hormone (α-MSH). The hormone α-MSH is an agonist of the melanocortin-4-receptors (MC4R), found in the paraventricular nucleus. The MC4R receptor has anorectic activity [6,12]. AgRP inhibits the MC4R agonist activity of melanocortin peptides (Figure 1a–c). In addition, a functional role in energy homeostasis for central and peripheral MC3R has been suggested [8]. In this system, hormones of the fed state (insulin and leptin) released by the pancreas and adipocytes (respectively) bind to their receptors on the POMC neurons and support the processing of proopiomelanocortin to α-MSH, promoting a signal to decrease food intake. Leptin also binds to AgRP /NPY neurons to inhibit their orexigenic activity. It is likely that leptin binding to neuronal cells other than the POMC neurons contributes to energy homeostasis [13] and that AgRP neurons engage other circuits that coordinate feeding. Axon projections of AgRP neurons extend to neurons in the bed nucleus of the stria terminalis, lateral hypothalamus, and parabrachial nuclei that control insulin sensitivity in brown adipose tissue. The axon projections of the AgRP neurons may trigger the motivational and autonomic circuits that contribute to feeding behavior. It is likely that multiple pathways function in the brain, one for homeostatic control of energy balance and feeding and another for non-homeostatic, reward-driven, hedonic feeding [13]. One suggestion is that the hedonic circuitry, once activated, can override the homeostatic energy balance circuitry and the chronic inhibition of AgRP [14]. The effects of lifestyle on epigenetic modifications of genes in the hypothalamus and its role in the regulation of energy homeostasis require further investigation [15].

Complex neurological and endocrine signals are relayed between the gut and the brain to regulate hunger and satiety. The gut neuroendocrine system and the gut-brain axis function to optimise digestion and absorption and regulate appetite. Numerous reciprocal connections exist between the brain stem (particularly the nucleus of the tractus solitaries) and the hypothalamus [16]. The brain stem receives vagus nerve afferents from the gastrointestinal tract and endocrine signals from the blood due to its closeness to the blood-brain barrier. It is positioned to act as a site of integration between endocrine and neuronal signals (Figure 1a). Afferent neurons of the vagus nerve targets for gut hormones [17].

## 4. The Endocrine System of the Gut and the Control of Appetite

Gut hormones are secreted by the enteroendocrine cells scattered throughout the epithelial cells of the gut. Gut hormones can act as both hormones and neurotransmitters. The incretin effect is described as the increase in insulin secretion by pancreatic β cells by exposure to intestinal absorption of glucose compared to isoglycemic levels by intravenous infusion. Gut derived hormones are the proglucagon-derived peptides, glucagon-like peptide 1 (GLP-1), GLP2, and gastric inhibitory peptide, also known as glucose-dependent insulinotropic polypeptide (GIP), as well as cholecystokinin (CCK) and peptide YY (PYY), released by the gastrointestinal tract into the general circulation, which mediate effects of feeding [18]. Both GIP and GLP-1, the real incretins, exert their effects by binding to their specific receptors, the GIP receptor (GIPR) and the GLP1 receptor (GLP1R), in pancreatic β cells and boosting the secretion of glucose-dependent insulin release [19,20]. Evidence points out that some enteroendocrine cells, which constitute approximately 1% of intestinal epithelial cells, are plurihormonal. In mice, GIP is secreted from K cells found predominantly in the duodenum, and GLP-1 is secreted from L cells located in the lower small intestine and colon. The presence of food (carbohydrates, protein, and fats) stimulates incretin hormone secretion. Similar to other gut peptides, GLP-1 is a neurotransmitter. Evidence for the involvement of GLP-1 in signalling in the CNS is the wide distribution of the GLP-1 receptor [21]. The GLP1R receptor is widely distributed in the body, for example, in the cardiovascular system, gastrointestinal tract, adipose tissue, brain, and bone [22]. Similarly, some studies report the GLP2R receptor to be widely expressed [23]. This highlights the diversity of incretin functions. A wide range of pharmacological drugs and pre-clinical studies have been used to clarify GLP1R/GIPR physiological function [24]. The suggested physiological roles for incretin hormones are summarised in Table 1.

### 4.1. Physiology of Incretin Secretion

Release of incretins (GLP-1 and GLP2 are cosecreted) requires the presence of nutrients, carbohydrates, proteins, and fat in the intestinal lumen and their absorption through the enterocytes [44]. To understand the process of altered incretin secretion following bariatric surgery, an understanding of the detailed molecular mechanism underlying its secretion is valuable and is included.

### 4.2. Secretion and Metabolism of Incretins

**Figure 2:**
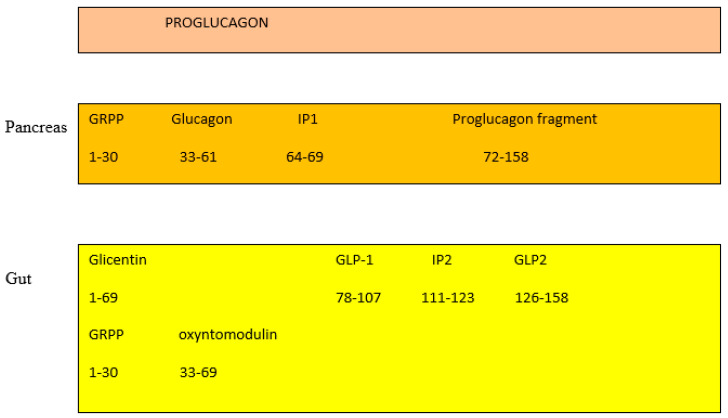
Gut and pancreas processing of the proglucagon molecule.

In the pancreas, the proglucagon molecule is processed by proconvertase 2 (PC2) to glucagon (Figure 2) and by proconvertase 1/3 (PC1/3) in the gut into GLP-1 (amino acids 1–37) and GLP2 (amino acids 1–33) [45]. Glucagon is secreted by the pancreatic α cell, stimulates glycogenolysis and gluconeogenesis in the liver, and opposes the hypoglycemic action of insulin. Bioactive GLP-1 (7–37) is generated from GLP-1 (1–37). Cleavage by PC1/3 leaves two basic residues at the C-terminus of GLP-1, which are removed by carboxypeptidase prior to carboxyamidation. Several immunoreactive forms of GLP-1 (including GLP-1 7–36 amide and GLP-1 7–37, which are thought to be equipotent), are released in vivo [46]. GLP-1 is inactivated by dipeptidyl peptidase IV (DPP-IV), which cleaves the N-terminal end. GLP-1 (7–36 amide) is thought to be the majority of circulating active GLP-1 in human plasma [47]. Degradation and inactivation of human GLP-2 (amino acids 1–33) with the enzyme DPP-IV resulted in the liberation of GLP-2 (amino acids 3–33) [48]. 

GIP is derived by proteolytic processing of a 153-residue precursor, preproGIP [49]. GIP is a 42 amino acid, and as it contains an alanine in position 2, it is physiologically degraded and inactivated by DPP-IV to its bioinactive form (amino acid 3–42) within minutes of secretion [50]. Both inactive GIP and GLP-1 are cleared by the kidney [51]. In healthy humans, the half-lives of GLP-1 is 2–3 min, GIP 5 min [52], and GLP-2 7 min [53]. Which of the two, GLP-1 or GIP, is the most prominent is still a matter of debate. Depending on the method protocol, it has been suggested that either GIP or GLP-1 contributes equally or that either GIP or GLP-1 contribute the majority of the incretin effect in humans [54]. One suggestion is that the contribution to the incretin effect is GIP: approximately 44%, GLP-1: approximately 22%, Glucose: approximately 33%, and neural transmission: negligible [55].

### 4.3. Control of Incretin Secretion by Nutrients

It has been shown that the presence of macronutrients (carbohydrates, proteins, and fats) in the gut regulates incretin hormone secretion.

#### 4.3.1. Carbohydrates

Glucose is a recognised stimulant of incretins and acts through the sodium glucose cotransporter 1 (SGLT1) with the influx of sodium ions, which depolarises the plasma membrane, opening the voltage-sensitive calcium channels, which is followed by exocytosis of GLP-1-containing secretory vesicles [56]. Facilitative glucose transport through the GLUT2 transporter appears to be of less importance in incretin secretion [57]. 

#### 4.3.2. Proteins

The pathways underlying the mechanism of protein stimulation of gut hormones are still not clearly understood. At the molecular level, meat hydrolysate has been shown to recruit the G-protein coupled calcium sensing receptor (CaSR) to cause GLP-1 release from the rodent intestine [58], though it is likely that other sensory pathways are involved, e.g., an increase in the anorectic hormone PYY [58,59].

#### 4.3.3. Fats

Fats also potentially stimulate CCK, PYY, and GLP-1 release. The release of CCK, PYY, and GLP-1 in plasma is attenuated by inhibitors of lipase. CCK, GLP-1, and PYY release were found to be dependent on fatty acid chain length, with only fatty acids greater than C10 being effective in stimulating hormone secretion [60,61]. Several G-protein-coupled receptors (GPCR) have been associated with the sensing of fatty acids and the release of gut hormones. GPR40 is a receptor for medium- and long-chain fatty acids. The distribution of GPR40 suggests it may act to regulate pancreatic islet and neurological cell function [62]. Close to the *GPR40* locus are the *GPR41* and *GPR43* genes encoding receptors activated by short-chain fatty acids [63]. GPCR receptors linked to GIP secretion are GPR120 and GPR40, expressed in K cells in the upper small intestine [64,65]. GPR40, GPR41, GPR43, GPR119, and GP120 were identified as sensing receptors for fatty acids with a role in GLP-1 secretion [66,67,68,69,70]. 

### 4.4. Other Variables That Regulate Gut Hormones

(i) Gut microbiome: Gut microbiota can contribute to incretin secretion either by fermentation of dietary fibre and production of short-chain fatty acids [71] or by increasing the basal level of GLP-1 and slowing intestinal movement [72].
(ii) Bile acids: Using a pharmacological approach, the bile acid-responsive receptor, G protein-coupled bile acid receptor 1 (GPBAR1/TGR5), has been shown to promote GLP-1 and PYY secretion from intestinal L-cells [73,74]. While bile acids are recognised to signal through specific nuclear acid receptors [75], GPBAR1 is a cell surface receptor. GPBAR1 is a GPCR that is activated by bile acids, resulting in stimulation of Gαs proteins and downstream cAMP signalling pathways. Recent findings support the hypothesis that additional pathways such as elevation of intracellular Ca^2+^ concentrations [76] and closure of ATP-sensitive potassium (KATP) channels may play a role in GLP-1 release [77]. Available data suggest that bile acids were more effective if they were applied to the basolateral GPBAR1 (vascular side) on L-cells and that luminal-applied bile acids are effective after absorption across the intestinal epithelial layer [78].
(iii) Proinflammatory cytokines TNFα, IL6, and Regulated on Activation, Normal T-cell Expressed and Secreted (RANTES) are increased in obesity in both rodents and humans. Acute treatment with TNFα increases GLP-1 release, and chronic elevation of TNFα decreases GLP-1 release. It has been suggested that TNFα alters cell signalling through the TNF receptor-NFκβ pathway [79]. IL6 regulates glucose homeostasis by stimulating L cells and pancreatic α cells to secrete GLP-1 and, as a result, increase insulin secretion. One suggestion is that acute effects of IL6 are caused by increased GLP-1 exocytosis in L cells and chronic effects by increased glucose responsiveness [80,81]. The chemokine RANTES reduced glucose and stimulated GLP-1 secretion in humans. One proposal is that RANTES acts through the CCR1 receptor to reduce cAMP levels and PKA activity [82]. The response of the L-cell is likely an integrated response to different cytokines using different signalling pathways.
(iv) Other suspected stimulants of GLP-1 secretion are progesterone (studies on cell lines suggest stimulation through the extracellular signal-related kinase 1/2 (ERK1/2) [83], insulin (studies on cell lines suggest a role for phosphatidylinositol 3 kinase-Akt and MAPK kinase (MEK)-ERK1/2 pathways [84], and glucocorticoid (reduced GLP-1 secretion in rodents) [85].

The contribution of each of these additional effects to incretin secretion is unclear, and further studies are required to understand the mechanism underlying these effects. 

## 5. Other Hormones Implicated in Appetite Regulation

The function of various hormones in regulating appetite and satiety to maintain energy homeostasis is included below.

### 5.1. Insulin

In the liver, insulin supports the transport of glucose from the blood to hepatocytes, where it is converted to glycogen, fatty acids, and triglycerides. In the skeletal muscle, insulin promotes the absorption of glucose. In adipose tissue, insulin supports the uptake of fatty acids, which are converted to triglycerides [86]. Insulin receptors are widely distributed throughout the CNS. Insulin has central anorexic effects. Insulin administration within the ARH can decrease the intake of food by reducing the activation of AgRP/NPY neurones and by increasing the activity of the POMC pathway. It has been proposed that insulin’s actions may be mediated additionally within non-hypothalamic regions [87].

### 5.2. Glucagon

Glucagon is a 29-amino acid peptide that is secreted from pancreatic α-cells in response to low levels of blood glucose. The glucagon receptor is a G-protein-coupled receptor that is mostly expressed in the liver with minor amounts in the brain and heart. Factors that regulate the secretion of glucagon are glucose, amino acids, gastrointestinally derived peptides, the autonomic nervous system, and possibly intra-islet regulation from beta cells and the inhibitory action of somatostatin. Glucagon secretion increases in hypoglycemic conditions with increased hepatic glucose production as a result of decreased glycogenesis and glycolysis and stimulation of glycogenolysis and gluconeogenesis. In the liver, glucagon can promote beta oxidation, decreased fatty acid synthesis, and decrease very low density lipoprotein release [88]. Early work suggests that glucagon contributes to satiety and that the satiety signal is transmitted through the vagus nerve [89]. A series of human studies confirmed the thermogenic effects of glucagon, an effect that was apparently dependent on the feeding state. One possibility is that the increase in energy expenditure is caused by the catabolic actions of glucagon and is not fully dependent on BAT [90]. Glucagon was thought to increase heart rate and contractility by acting through glucagon receptors and increasing cAMP, though recent results provide conflicting evidence [91,92].

### 5.3. Ghrelin

Ghrelin is an orexigenic hormone. The human ghrelin gene is localised on chromosome 3p25-26. Ghrelin mRNA is mostly expressed in gastric tissue, and it is also present at low levels in the intestine, pancreas, kidneys, and placenta. PC 1/3 processes proghrelin to ghrelin with acyl modification of Ser3 in ghrelin by the enzyme ghrelin O-acyltransferase. Preprandial rise and postprandial fall in plasma ghrelin levels support the hypothesis that ghrelin plays a physiological role in meal initiation in humans [93]. Ghrelin is an endogenous ligand of the growth hormone secretagogue receptor type 1a (GHS-R1a), which stimulates growth hormone (GH) secretion. GHS-R1a is a GPCR receptor. GHS-R1a is expressed in the hypothalamic and exra-hypothalamic regions and metabolic organs. GHS-R1a interacts with Gα_q/11_ and the phospholipase C and inositol triphosphate pathways. GHS-Rs are expressed in AgRP/NPY neurons and POMC neurons. The complexity of the role of ghrelin-mediated signalling in the arcuate nucleus and ventromedial nucleus of the hypothalamus are described in the review by Yanagi et al. [94]. However, studies with ghrelin KO mice models have shown that the mice have no decrease in appetite or appreciable difference in body weight, and it has been suggested that ghrelin may play a role during extreme nutritional challenges to protect bodyweight and has a further function in regulating blood glucose [95]. Ghrelin may act to relay meal-related information; ghrelin increases in preference for highly palatable food and may play a role in stress-induced food consumption, which results in hedonic feeding [96].

### 5.4. Secretin

Secretin is secreted by the enteroendocrine S-cells in the proximal duodenum in response to acidic chyme from the stomach. Both secretin and its receptor are ubiquitously distributed throughout the body, including the central nervous system. Secretin is associated with (i) stimulation of pancreatic water and bicarbonate secretion; (ii) body fluid homeostasis; and (iii) lipolytic actions in mouse adipocytes [97]. The half-life of secretin is approximately 2.5 min [98]. In mice, secretin and its receptor have been identified in ARH and the paraventricular nucleus, suggesting a central role for secretin in the control of appetite [99]. In humans (and mice), secretin activates (increased metabolism, thermogenesis) brown adipose tissue (BAT) and delays the central nervous system response to appetising food, and the impulse to refeed after a meal, and stimulates satiation [100,101]. However, secretin receptor KO mice on the same diet gained significantly less weight and had lower body fat content than their WT counterparts, suggesting further work is required on the role of the gut-BAT-brain axis in the role of chronic energy balance [102]. Secretin has been shown to have pleiotropic effects, and secretin receptors have been identified in the heart and kidney. Secretin has been shown to increase myocardial glucose uptake and increase renal filtration [103].

### 5.5. PYY, PP, and NPY

All three are members of the neuropeptide Y (NPY) family and have an amidated carboxyl terminus with a hairpin fold referred to as the PP fold, the latter required for the function of the NPY family. Peptide YY (PYY) hormone is primarily secreted by the L cells of the gut; there are two main forms of the hormone: PYY_1–36_ and PYY_3–36_, the latter created by DPP-IV by cleavage of the N-terminal amino acid residues. PYY immunoreactivity is also found within the central nervous system. There are 5Y receptors (Y1 to Y5, members of the G-protein-coupled receptor family), and PYY_3–36_ the major form in circulation, has a high affinity for Y2 and a lesser affinity for Y1 and Y5 receptors. In the rat, Y2 mRNA has been detected in the central nervous system [104]. The Y receptors are widely distributed in the central and peripheral tissues. Each Y receptor has a varying distribution across central and peripheral tissues [105,106]. Basal plasma PYY levels are low but rise in response to food, and postprandially they remain elevated for several hours. [107]. A high protein diet causes the greatest satiety and PYY release in humans [59]. Intravenous PYY_3–36_ decreased appetite in both healthy and obese subjects [108]. It has been suggested that PYY_3–36_ acts by modulating the activity of NPY and POMC neurons in the ARH of the hypothalamus [109], to decrease food intake. Other studies suggest that peripheral PYY_3–36_ may conduct satiety signals to the brain by the vagal afferent pathway [110]. 

Pancreatic polypeptide (PP) is a 36-amino acid peptide secreted by specialised pancreatic islet cells called F cells, and a small amount is found in the gut. Release of PP is postprandial and can remain elevated for up to 6 h following food intake [111]. PP, similar to PYY, is a satiety hormone, and though it binds all members of the Y receptor, it exhibits greater affinity for the Y4 receptor [112]. Investigations suggest possible mechanisms for PP-induced satiety are (i) delay in gastric emptying and increasing energy expenditure [113]; (ii) anorectic actions through increasing the activity of POMC neurons. The latter effect is thought to be a direct consequence of the activation of Y4 signalling [114]. Other data suggest that PP may regulate food intake by suppressing the orexigenic pathways by downregulating the orexigenic hormone orexin and simultaneously increasing the anorexigenic pathways by upregulating the anorexigenic brain-derived neurotrophic factor [115]. Following vagotomy, PP does not exert its anorectic effects, suggesting its function may depend on the vagus nerve [113]. 

The neuropeptide NPY, a 36 amino acid peptide, is a member of the NPY family and is abundantly expressed in the central and peripheral nervous systems. NPY is a member of the NPY family and is included in this section for completeness. NPY is found in the postganglionic sympathetic neurons, adrenal medulla, and peripheral tissues such as adipose tissue, pancreas, liver, skeletal muscle, and osteoblasts [116]. NPY is an orexigenic neuropeptide. NPY and PYY have identical affinities for the Y receptor [117]. In the central nervous system, the highest concentration of NPY is found in the ARH. The NPY receptors are found in many hypothalamic neurons, connecting the limbic and autonomic nervous systems with the hypothalamus [118]. It has been suggested that NPY has a role in decreasing the browning of white adipose tissue and thermogenesis in brown adipose tissue [119].

### 5.6. Cholecystokinin (CCK)

CCK producing cells are located primarily in the proximal small intestine in I cells. Studies suggest that the cells contained axon-like basal projections called neuropods. One hypothesis is that the axon-like processes drive hormone secretion and that hormone secretion is near enteric nerve cells [120]. CCK in plasma is a heterogeneous mixture of medium-sized peptides processed from the primary translational product preproCCK (115 aminoacids): CCK-58, CCK-33, CCK-22, CCK-8, and CCK-5, and the Y-77 site is mostly O-sulfated. In addition, CCK is a carboxyamidated peptide. CCK has been reported to be widely expressed in mammalian tissue, intestinal tissue, the cerebral and peripheral nervous system, extraintestinal endocrine glands, the urogenital tract, and the cardiovascular system [121]. In human plasma, using well characterised assays, Rehfeld [122] et al. showed that CCK-33 and CCK-22 were the most abundant and CCK-58 less abundant in human plasma, though there was significant species variation. Fasting levels of CCK averaged 1.0 pM and rose to 6 pM within 15 min following ingestion of a meal. Fat, protein, and amino acids were all stimulators of CCK secretion in contrast, glucose caused a smaller elevation in CCK levels [123]. In dogs, the half-life of CCK-58 was approximately 4 min and that of CCK-8 was 1.3 min, suggesting a short half-life for CCK [124]. The actions of CCK are mediated by two G-protein-coupled receptors, CCK1 and CCK2. CCK1 receptors are found in the gastrointestinal tract, myenteric plexus, and vagal afferents. CCK1 binds sulfated CCK with a higher affinity than non-sulfated CCK. CCK2 receptors are present in the stomach and brain and have similar affinities for sulfated and non-sulfated CCK [121].

Longer (CCK-58, CCK-33) as well as shorter forms are expressed in cerebral neurons and are neuronal peptides and potent neurotransmitters [125]. CCK interacts with CCK1 receptors localised in specialised regions of the hindbrain to induce satiety. In rat studies, CCK has been shown to inhibit the expression of orexigenic peptides (e.g., orexin [126]) in the hypothalamus and prevent stimulation by ghrelin [127]. When plasma CCK concentrations are low, vagal afferent neurons are associated with stimulation of food intake, while increased postprandial CCK vagal afferent neurons are linked to downregulation of food intake [128]. Other effects of CCK are to (i) stimulate exocrine pancreatic secretion, (ii) induce gall bladder contraction, and (iii) delay gastric emptying [129].

### 5.7. Somatostatin

Endocrine cell-secreting somatostatin (SST)-secreting cells are found in the stomach, intestine, and pancreas. SST is a cyclic oligonucleopeptide, with two forms of 14 aa and 28 aa. SST has five types of G-protein-coupled SST receptors (SSTR1 to SSTR5 and two isoforms SSTR2A and SSTR2B). About 5% of pancreatic cells are SST-secreting δ cells. SST is secreted in response to hypoglycaemia. Somatostatin secreted within the pancreas is a paracrine inhibitor of glucagon and insulin secretion [130].

About 90% of SST is located in the GIT D endocrinal cells, and 10% is located in the enteric nervous structures. One estimate is that GIT D cells contain 65% of the total body SST, the pancreatic cells 5%, and the rest in the CNS [130,131]. SST inhibits parietal cells, ghrelin cells, and gastrin through paracrine inhibition [131]. Removal of this restraint during ingestion of food increases acid and gastrin secretion [132]. SST has been shown to have a widespread distribution in the central nervous system of rodents and humans. Pleiotropic effects of brain-initiated SST have been described, and this includes GIT motility. In rodent studies, centrally injected SST stimulated food intake, though contradictory findings suggest that SST has an anorexigenic effect [133]. A summary of hormones and their functions in energy homeostasis are summarised in Table 2. 

## 6. Adipocyte Hormones

Adipocyte tissue is formed by mature adipocytes, fibroblasts, endothelial cells, adipocyte progenitors, and immune cells. Three main types of adipocyte exist in humans white, brown, and beige. White adipocytes can store energy, whereas mitochondria-rich brown adipocytes dissipate energy by thermogenesis. Both cell types are found in multiple fat depots forming the adipose organs. Adipocytes are also endocrine organs secreting adipokines, batokines (from brown adipose tissue), lipokines, microRNA, and inflammatory cytokines [153,154]. Leptin and adiponectin are the most known secretory products. Other adipose tissue secretory products have been reviewed extensively [153]. Beige adipocytes can also dissipate energy. White-to-brown conversion is in response to certain stimuli, such as chronic cold exposure or in response to β3 adrenergic stimulation [155]. In recent years, it has emerged that white, brown, and beige adipose tissue play a role in the regulation of metabolic health through the secretion of several adipokines. In addition, adipocytes respond to hormones to carry out lipogenesis and lipolysis. Due to these functions, adipose tissue is considered a vital regulator of metabolic homeostasis. 

### 6.1. Leptin

Leptin is a 167 aa peptide that is primarily secreted by the adipose tissue. It is also found in several other tissues, including the lymphoid tissue [156]. Leptin is secreted in a circadian rhythm, with low levels at mid-afternoon and highest level at midnight. Levels increase when adipose tissue mass increases; decreasing food intake and weight loss lead to a decrease in leptin levels and consequently an increase in food intake. This serves to control the adipose tissue mass within a narrow range by linking the changes in energy stores and physiological responses. The principal site of action for leptin is the brain. Further sites established outside the brain for the direct action of leptin are the immune cells. Factors stated as controlling leptin secretion are insulin, glucocorticoids, and catecholamines [157]. Controversy exists over whether exercise has an effect on leptin levels [158,159]. In rodent studies, there is a cold-induced suppression of leptin gene expression in WAT, and it is likely that this is mediated by the sympathetic nervous system [160].

Leptin receptors (LepR) are in the class 1 cytokine receptor family with a single membrane spanning domain. Several isoforms of the receptor (LepRb is the longest) have been identified. These isoforms have homologous extracellular binding domains, but their intracellular domains vary in length due to alternative splicing. Leptin receptors activate several transduction pathways. The pathways include Janus kinase 2 (JAK2), signal transducer and activator of transcription 3 (STAT3), insulin receptor substrate (IRS)-phosphatidylinositol 3-kinase (PI3K), SH2-containing protein tyrosine phosphatase 2 (SHP2)-mitogen-activated protein kinase (MAPK), and 5’ adenosine monophosphate activated protein kinase (AMPK)/acetyl-CoA carboxylase (ACC), as well as other pathways [2]. LepR is widely distributed in several peripheral tissues. This raises questions about the role of leptin in these tissues. Leptin appears to have a range of roles in the peripheral tissues, which include the pancreas, liver, skeletal muscle, adipose tissues, immune cells, and cardiovascular system. Its effect may depend on whether leptin is acting centrally or directly on peripheral tissues [161]. Leptin is increased in obesity, and one of the major risk factors for obesity is leptin resistance, which leads to a decrease in satiety caused by disruption of signalling pathways of leptin. The changes in signalling pathways are triggered by inflammation [151]. 

In the ARH, leptin decreases food intake by activating POMC and inhibiting AgRP/NPY neurons. During fasting, the fall in leptin stimulates AgRP/NPY neurons and suppresses POMC to increase food intake and decrease energy expenditure [2]. In rats, leptin increased sympathetic nerve activity in the BAT and adrenal glands. Sympathetic activation of BAT would be expected to enhance thermogenesis [162]. One suggested mechanism is through increased expression of the uncoupling protein and increased thermogenesis [163]. Rodent studies suggest that leptin and insulin can act synergistically to promote WAT browning by acting on POMC [164]. The coupling of leptin levels to adaptations in food intake and energy expenditure enables long term weight maintenance. It has been stated that the organism’s sense of nutritional state (adipose tissue mass) is conveyed by leptin [165].

### 6.2. Adiponectin

**Figure 3:**
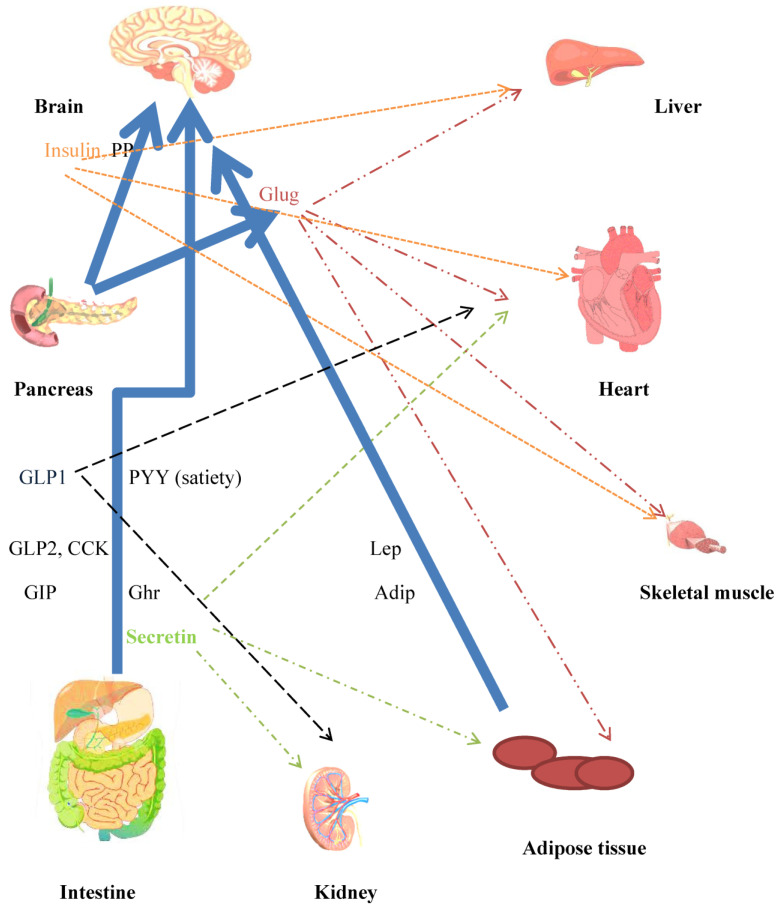
Crosstalk between peripheral and central mechanisms that contribute towards whole body energy homeostasis. Major mediators of mechanisms for controlling energy homeostasis. Neuroendocrine hormones are released from the gut in response to nutrient intake. Incretins released from the gut have several effects, in addition to increasing insulin secretion. The CNS is a target for the anorexigenic/orexigenic and satiety effects of the hormones. Blue unbroken arrows show routes for hormone secretion. Effects on peripheral tissues, heart liver, skeletal muscle, and adipose tissue are indicated by broken arrow lines, color coded to agree with hormones. Hepatocytes and adipocytes are implicated in glucose/lipid and amino acid metabolism. Adipocytes secrete the adipokines leptin and adiponectin. Insulin, leptin, and secretin are hormones concerned in WAT browning and BAT thermogenesis. CCK (decreases food intake), Lep: leptin (anorexigenic), Adip: adiponectin (orexigenic), Ghr: ghrelin (orexigenic), Sec: secretin (satiety hormone), Glug: glucagon.

Adiponectin is mainly synthesised in adipocytes. The monomeric protein is postranslationally modified into different multimers. The plasma adiponectin half-life was 2.5 h. Unlike other adipokines, adiponectin blood concentrations are inversely associated with fat mass. Weight loss due to caloric restriction in obese humans increases adiponectin tissue gene expression and plasma concentrations towards normal lean levels. Adiponectin increases insulin sensitivity and decreases hepatic glucose output. AdipoR1 and AdipoR2 were identified as receptors for adiponectin. Both receptors are expressed widely, though AdipoR1 is more highly expressed in skeletal muscle and AdipoR2 is more restricted to the liver. Adiponectin’s insulin sensitising actions are mainly in the liver and skeletal muscle [166]. In mice, adiponectin decreases glucose production in the liver [167] and increases fatty acid oxidation in rodent skeletal muscle [168]. Transgenic mice lacking leptin but overexpressing adiponectin had significantly higher levels of adipose tissue, suggesting that adiponectin promoted the storage of triglycerides in the adipose tissue [169]. AdipoR1 and AdipoR2 are expressed in human and rat pancreatic cells [170]. Studies on human pancreatic cells suggest that, in addition to its anti-apoptotic action, adiponectin has another effect on beta cells by potentiating insulin secretion [171]. Chronic cold exposure led to an elevated production of adiponectin in subcutaneous WAT. Adiponectin knockout mice showed blunted browning of subcutaneous adipose tissue in response to cold exposure, suggesting a role for adiponectin in cold-induced browning of subcutaneous WAT [172]. Studies on mice suggest that adiponectin is an orexigenic hormone and that it may stimulate the phosphorylation of AMP-activated protein kinase in the ARH [173]. Adiponectin is also an anti-inflammatory agent and, as a result, has protective effects on the vasculature, lung, heart, and colon [166]. The role of several hormones in energy homeostasis is summarised in Figure 3. 

## 7. Genetic Mutations That Affect Energy Homeostasis

Individual contributions to mechanisms that control energy homeostasis are illustrated in mutations that affect metabolic function. Monogenic obesity has contributed to the understanding of the process of energy/metabolic homeostasis.

(i) POMC mutations: patients with complete loss of POMC gene function were diagnosed on the basis of secondary hypocortisolism, red hair, and extreme obesity [174].
(ii) Leptin and leptin receptor mutations: associated with morbidly obese phenotypes and disturbances in metabolic and immune functions [175].
(iii) MC4R mutations are linked to severe obesity [176].
(iv) Ghrelin receptor mutations: patients present with short stature and obesity [177].
(v) Loss of function of *PCSK1* variants (encoding prohormone convertase subtilisin/kexin type 1) with a deleterious effect on PC1/3 proconveratse activity contributes to obesity [178].
(vi) NPY-variants within the gene are associated with obesity [179].
(vii) Brain-derived neutrotropic factor (BDNF, a neurotropin) and its receptor tropomyosin receptor kinase B (Trkb) have been shown to act in the brain to regulate neuronal survival, growth, and plasticity. BDNF and TrkB regulate several physiological processes. BDNF has been suggested to play a role in weight loss, energy expenditure, and thermogenesis. Mutations in TrkB have been associated with obesity [180].
(viii) Polygenic forms of obesity: genome wide association studies (GWAS) and next-generation sequencing (NGS) have increased awareness of the polygenic forms of obesity. GWAS studies have suggested that the FTO gene (widely expressed in tissues and the brain) is a significant genome contributing to obesity. A total of 127 sites in the human gene have been linked to obesity through GWAS studies. Some of the genes implicated in the development of obesity are: uncoupling proteins in the brown adipose tissue, the *SLC6A14* gene, a transporter of amino acids [181].
(ix) Syndromic forms of obesity have been reviewed [182]. Some examples are: Prader–Willi syndrome (PWS), Down syndrome, Bardet–Biedl syndrome, fragile X syndrome, Alstrom syndrome, and Cornelia de Lange syndrome.

## 8. Bariatric Surgery

**Figure 4:**
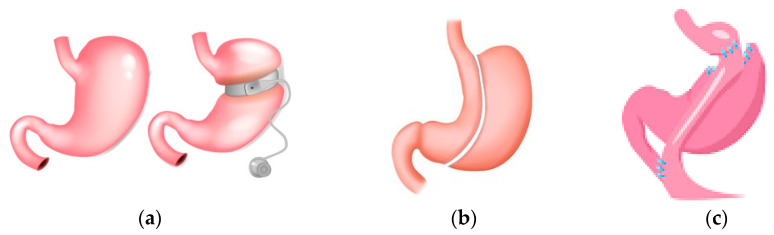
Surgical procedures common in bariatic surgery. (**a**) Gastric banding: A silicone band that is adjustable is placed below the gastroesophageal junction; this suppresses hunger. The level of restriction can be adjusted by the amount of fluid in the band. It is a reversible method with the lowest morbidity and mortality. Disadvantages are gastric band leak, gastric band dilation, and the highest reoperation rate. (**b**) Gastric sleeve: Gastric volume is reduced by the removal of a large portion of the stomach. The disadvantages are staple line leaks, gastroesophageal reflux disease and dilation of the remaining gastric remnant. (**c**) Gastric bypass (RYGB): Gastric volume is reduced, and the small gastric pouch created is joined to the jejunum. Nutrients flow from the gastric pouch to the proximal jejunum, with bypassing of the lower stomach and duodenum. Disadvantages are staple line leaks, dumping syndrome, and gallstones.

Obesity is considered a chronic disease that increases morbidity and mortality. Bariatric surgery (surgical procedures for obesity treatment—referred to interchangeably as bariatric surgery, weight loss surgery, metabolic surgery, or metabolic/bariatric surgery) has been shown to be effective in achieving weight loss, and studies show a lower mortality compared to control subjects under non-surgical conventional treatment [183,184]. Three common forms of bariatric surgery are sleeve gastrectomy (SG), adjustable gastric band (AGB), and Roux-en-Y-gastric bypass (RYGB). The surgical procedures with advantages and disadvantages are summarised in Figure 4 [185]. Guidelines suggest that bariatric surgery should be considered in individuals with BMI ≥ 40 kg/m^2^ or BMI ≥ 35–40 kg/m^2^ with co-morbidities [185,186,187] that are anticipated to improve with weight loss. They further address optimised care before, during, and following surgery [185,186,187], https://www.nice.org.uk/guidance/cg189 (accessed on 23 February 2024). Patients following bariatric surgery are required to have nutritional surveillance and laboratory screening for nutritional deficiencies. In the Swedish Obese Subjects study, surgically treated patients had greater weight loss, more physical activity, and decreased energy intake compared to control subjects [188]. One guideline mentions a review for monogenic and syndromic forms of obesity if historical and physical findings require further investigations and decisions made on a case by case basis [185]. For polygenic obesity, common variants have been identified; each variant has a small effect on body BMI, with interaction with the environment playing a key role. Variants in or near genes that result in severe obesity may have a more subtle impact on individual BMI. Patient genotype may allow a more exact understanding of patient obesity and permit a more exact treatment [189].

Briefly, bariatric surgery addresses health and quality of life when lifestyle changes cannot decrease weight adequately to prevent complications associated with obesity. However, bariatric surgery has to be balanced alongside the risks associated with surgery and consequent mortality/morbidity, prospective nutritional deficiency, weight regain, and the need for lifelong support and medical monitoring. Nutrition surveillance includes iron, folate, vitamin B12, vitamin D, calcium, fat-soluble vitamins, and trace elements [190].

### 8.1. Bariatric Surgery and Absorption

Following surgery, the gastrointestinal physiology is altered. Mechanisms that lead to weight loss are still incompletely understood. Gastric pouch emptying is altered, though further studies are required, including the variables gastric pouch size and emptying and meal size and texture [191]. This may lead to changes in the postoperative patient driven by changes in diet, changes in anatomy and physiology of the GIT tract, which may be based on the type of procedure performed and may lead to changes in the digestion and absorption of food and changes in nutritional state. Changes in gastric microbiota are found in the obese state compared to lean microbiota composition. Alterations in the microbiota of obese patients following bariatric surgery have been observed. Accelerated gastric transit time and decreased gastric acid production can contribute to changes in gut microbiota. The extent to which gut microbiota affect the host BMI is difficult to determine [192]. Both RYGB and SG have been shown to increase systemic bile acid concentrations in the circulation. One hypothesis is that the bile acid receptor TGR5 shows an increased response to elevations of bile acid and may play a role in GLP-1 secretion [193].

Physiological changes following bariatric surgery are still to be investigated extensively. Studies have either investigated SG and RYGB patients separately during ongoing weight loss, during the weight stable phase, or studies lacked a control group. There are multifactorial mechanisms affecting weight loss following RYGB/SG surgery. The favourable effects are not caused by malabsorption and food restriction alone but also by changes in GIT hormones. 

### 8.2. Gastrin

Gastrin levels were reported to fall following RYGB, increase during GS, and show no change during AGB. Histological changes associated with RYGB are atrophic gastritis and chronic gastritis, and patients are given proton pump inhibitors postoperatively [194].

### 8.3. Ghrelin

It has been shown that ghrelin levels fall following RYGB and GS, though studies suggest a long term increase in ghrelin levels following RYGB in some patients [194,195]. Peterli et al. [196] report that in patients following GS, ghrelin showed decreased levels at 1 year, though slightly higher than at 3 months.

### 8.4. Cholecytokinin

Studies have shown an increase in CCK levels postprandially after RYGB; in one study, GS showed a higher response in CCK than RYGB [194]. 

### 8.5. GLP-1

Studies suggest an increase in the satiety hormones GLP-1 and PYY, as well as oxyntomodulin, following RYGB. One suggestion is that the rise in gut hormones in RYGB can be related to the rate of pouch emptying and the direct delivery of nutrients into the proximal jejunum. Increased satiety hormones appear to play a role in optimal weight loss. Postprandial GLP-1 has been shown to be elevated compared to pre-operative levels at different time points post-surgery [195]. Increases in GLP-1 and PYY are also seen following SG [196].

### 8.6. GLP2

Postprandial GLP2 levels have been shown to increase following RYGB surgery, though no significant differences were demonstrated between fasting levels in preoperative and postoperative patients [195]. Cazzo et al. [197] suggest an increase in GLP2 following SG. Further investigations on the effects of bariatric surgery on GLP2 are required. 

### 8.7. PYY

Postprandial plasma levels of PYY rise in patients following RYGB, though reports of increased PYY are not always consistent, suggesting further work is desirable [195]. An increase in PYY has been shown to occur following AGB, GS, and RYGB [194]. In a comparison of good versus poor responders in RYGB for weight loss, suboptimal PYY and GLP-1 postprandial responses were associated with poor responses [198].

### 8.8. GIP

In a meta-analysis, Gao et al. [199] state that fasting GIP levels are significantly reduced following RYGB surgery and that the decrease was more pronounced in diabetic subjects. They acknowledge the need for randomised prospective studies with larger sample sizes to confirm these findings.

### 8.9. PP

PP has been generally reported as unchanged after RYGB and SG, though some studies have reported lower fasting levels post-RYGB [200].

### 8.10. Adipokines

Following both RYGB and GS, adiponectin levels increased and leptin levels decreased [201]. This was paralleled by improved insulin sensitivity and normalisation of lipid profiles [202].

## 9. Gut Hormone Changes after Metabolic Bariatric Surgery and Their Contribution to Weight Loss

The alterations in gut hormone levels following bariatric surgery have been suggested as an important mediator of eating behaviours favouring weight loss in the long term. The main changes in gut hormones and adipokines are shown in Table 3. The increase in GLP-1 and PYY is thought to increase satiety and reduce hunger. Studies suggest that postprandial glucose and protein absorption and gut hormone secretions differ between SG and RYGB. RYGB was characterised by increased absorption of glucose and amino acids, whereas protein metabolism after SG did not differ very significantly from controls. In one study, there was a marked difference in gastric pouch emptying between SG and RYGB. The authors suggest that diverging rates of intestinal nutrient entry and absorption may explain the differences in the secretion of GIT hormones [203]. Other studies confirm the higher postprandial levels of GLP-1 following RYGB compared to SG [204], though other authors report a lack of difference in GLP-1 secretion between RYGB and SG [205]. The differences between studies can be attributed to various factors, including differences in surgical techniques and different limb lengths in RYGB. The role of post-surgical vagal nerve fibres in alterations to changes in food intake and GIT hormone secretion has not been completely investigated [206].

It has been suggested that each bariatric procedure has its own unique hormone profile. Differences in the rate of gastric pouch emptying, food intake restriction, and changes in the gut hormone profile may play a synergistic role in increasing satiety and reducing post-operative food intake. A few studies suggest that increased postprandial GLP-1 and PYY secretion 6 years after RYGB was associated with increased weight loss. The causality of the association has been queried, and it has been suggested that it has to be interpreted in the context of other hormone levels, e.g., a reduction in insulin and leptin levels. Studies that report an increase in fasting ghrelin following weight loss suggest that ghrelin changes are a consequence, not a cause of weight loss [207]. A study using the blockade of the actions of both GLP-1 and PYY suggests that both hormones are involved in inhibiting food intake following RYGB [208].

## 10. Postprandial Hypoglycaemia following Surgery

Postprandial hyperinsulinemic hypoglycaemia is a complication of RYGB and GS [209]. Hypoglycaemia may accompany dumping syndrome [210], though dumping syndrome can often be missed [211]. Studies further suggest that postprandial hypoglycaemia is not exclusively postprandial [212]. Symptoms of hypoglycaemia can vary from mild (sweating, weakness, and dizziness) to more severe neuroglycopenic symptoms, which include seizures, coma, and loss of consciousness [213]. 

Several hypotheses have been put forward to explain the pathophysiology of post-bariatric surgery hypoglycaemia

(i) Rapid gastric emptying, which results in a steep rise in plasma glucose compared to non-surgical controls [214].
(ii) Studies that used GLP-1R antagonists corrected the underlying hypoglycaemia in symptomatic patients, suggesting a role for GLP-1 in the underlying pathophysiology [213]. In other studies following RYGB individuals with post-RYGB hypoglycaemia had greater postprandial GLP-1 and insulin concentrations but not GIP when compared with individuals who had the same bariatric surgery but did not develop hypoglycaemia [215]. One hypothesis, supported by cross-sectional studies, is that postprandial hyperinsulinemia after RYGB can be attributed to the combined effects of more rapid nutrient transit from the gastric pouch to the gut coupled with an increased incretin effect [214,216]. One study reported decreased insulin clearance in the latter portion of postprandial hypoglycaemia contributed to relative hyperinsulinemia. A multifactorial model for the glucose dysregulation in postprandial hypoglycaemia has been suggested [217].
(iii) The possibility of other factors that may influence glucose metabolism has been suggested: reduced counter-regulatory hormone response, increased insulin sensitivity following weight loss, altered bile acid metabolism [214].

Various treatment modalities have been proposed and are covered in detail elsewhere, and a summary will be included in this review: (i) dietary modification to include complex carbohydrates; (ii) medical treatment with acarbose, octreotide, diazoxide, and calcium channel blockers; (iii) surgical treatment, latter only for severe refractory hypoglycaemia. Although the use of GLP-1 receptor agonists is counterintuitive, authors have suggested its use if other alternatives are not successful, though further work is needed prior to the use of these drugs [218]. 

## 11. Weight Regain following Bariatric Surgery

Following bariatric surgery, it is now accepted that a proportion of patients incur weight gain during long term follow-up. The reported prevalence of weight gain will depend on the definition and time since surgery. One study reports that during time points from 2–5 years, the weight regain fluctuated from 2.53–94.18% depending on the definition of weight regain [219]. 

Weight loss due to a low calorie diet results in significant reductions in levels of leptin, PYY, CCK amylin, and insulin and increases in levels of ghrelin, GIP, and PP [220]. PYY, GLP-1, CCK, PP and amylin inhibit food intake, while ghrelin stimulates hunger. In contrast, following bariatric surgery, exclusion of the foregut leads to an increase in hormones that promote satiety as well as downregulation of ghrelin, which stimulates food intake [221]. However, there is controversy over the role of appetite related hormones in weight loss relapse [222]. Additional lifestyle modifications that change eating habits and physical activity can improve weight loss outcomes [223]. 

A case series with rare biallelic mutations in the leptin-melanocortin pathway (leptin, MC4R, and POMC genes) showed a limited benefit of bariatric surgery [224]. Cooiman et al., [225], suggest that heterozygous mutations in POMC and PCSK1 do not affect the effectiveness of bariatric surgery in the first two years of follow-up. Other authors confirm that carriers of a heterozygous variant in the leptin-melanocortin pathway have a progressive and significant weight regain in the mid- and long-term after RYGB [226]. Studies that combine several SNPs suggest that weight loss and long term weight regain after bariatric surgery may be influenced by multiple genetic variants [227]. A combination of several genes and the development of a genetic risk score may have a predictive value for an individual’s predisposition to obesity [228]. Bonetti et al. [229], suggest that a next-generation sequencing panel (NGS), which include diagnostic and candidate genes, could play a role in predicting the outcomes of bariatric surgery.

In a recent review, van der Meer et al. [230] suggest that there is limited evidence supporting a role for genetic variants in outcomes following bariatric surgery. They suggest that more evidence and studies are needed to replicate findings across sexes and ethnicities. Identifying carriers of deleterious genes that may lead to suboptimal short term weight loss or long term weight regain might help implement individualised therapies that take into account impaired genetic pathways that are less functional in these individuals. Early genetic evaluation can identify treatable obesity and establish more personalised management.

The recent development of therapeutic options for the treatment of genetic obesity has increased treatment alternatives for genetic obesity other than lifestyle management and bariatric surgery. Options available are (i) GLP-1 analogues, (ii) MC4R agonists, and (iii) recombinant leptin. These drugs are reviewed extensively elsewhere [12,231].

## 12. Type 2 Diabetes Mellitus (T2DM) and Bariatric Surgery

Obesity is a risk factor for T2DM as well as cardiovascular disease, musculoskeletal disease, and many cancers [232]. Bariatric surgery and weight loss can lead to T2DM remission [233]. Surgery has been shown to be more effective than medical treatment for long term control of T2DM in obese patients. The authors suggest continued monitoring of patients as there is a potential relapse of hyperglycemia [234]. Population-based data show that bariatric surgery, RYGB and GS, increases the chance of remission of T2DM, though the risks of surgery have to be balanced against its benefits [235]. Patient compliance with lifestyle modifications post-bariatric surgery is linked to weight loss outcomes and comorbidity resolution [236]. Following bariatric surgery, markedly enhanced insulin secretion is observed. This has been explained by a rapid transit of nutrients to the distal part of the small intestine and an increased postprandial secretion of GLP-1 [237]. Both weight loss-dependent [238] and weight loss-independent [239] changes have been hypothesised to yield improvements in glucose metabolism. Postprandial increases in GLP-1 [240] and (in rat studies) improved pancreatic β cell function following RYGB can contribute to T2D remission [241]. Mice studies suggest an improvement in hepatic and muscle insulin sensitivity following SG [242]. It has been hypothesised that gut microbiome changes and bile acid signalling post-bariatric surgery may lead to improved insulin sensitivity [243].

Relapse of diabetes mellitus can occur following bariatric surgery and treatment with anti-diabetic drugs; sodium-glucose cotransporter 2 inhibitors [244] and GLP-1 receptor agonists are options to prolong diabetes remission or extend treatment in patients with less effective glycemic control [245]. These medications have been reviewed in previous articles [244,245].

## 13. Conclusions

Several interconnected neural circuits in the brain control both energy needs and feeding behaviours. Neurons that respond to satiety are the POMC neurons that produce α-MSH, which binds to MCR4 receptors to decrease food intake. Conversely, decreased food intake triggers NPY/AgRP neurons to increase inhibition of POMC neurons, inhibition of α-MSH, and an increase in feeding response. The neurons are located in the ARH, which is considered an important centre for the integration of hunger and satiety [14]. The brain, mainly the hypothalamus, integrates the gut-brain axis signals, which are the many gut peptides that influence metabolic homeostasis. Neural signalling occurs through the vagus afferent neurons. Intestinal microbiota can further influence the gut-brain axis [246]. Obesity remains a complex, multifactorial disease that is now a major public health issue. Bariatric surgery remains an effective treatment with weight loss and metabolic benefits. A better understanding of the mechanisms and benefits associated with bariatric surgery is crucial for future pharmacological interventions in patients with morbid obesity. Replicating gut hormone changes through pharmacological means holds promise for future medical interventions in bariatric surgery. Further studies are needed to clarify the changes in pathophysiological pathways as a consequence of bariatric surgery. Knowledge of patients genotypes may allow for a more personalised treatment of obesity or the establishment of prevention strategies. Understanding the genetics of obesity may lead us towards new therapeutic targets and a more personalised and precise medicine for obesity. Multimodal treatments that include lifestyle management, bariatric surgery, and pharmacological treatments can be routes for obesity management. Combination co-agonist treatments and clinical trials are in progress [247]. The physiology of food consumption, weight control, and energy expenditure may be required for a more successful integrated understanding of obesity and its treatment in the future.

## Figures and Tables

**Table 1:** Functions of incretin hormones.

	GLP-1	GLP2	GIP	Possible Mechanism of Action	Reference
Source	L cells	L cells	K cells		
Brain	Decreases appetite and food intake and increases satiety	Found in areas of the brain involved in appetite regulation	GIP receptors gene cloned from the rodent CNS. It has been suggested that GIP may reduce food intake and improve cognitive function (studies carried out on rodent models)	(i) POMC neurons activated and AgRP neurons inhibited by GLP1R (ii) GLP-1 acts as a neurotransmitter	[25,26,27,28,29]
Heart	Multiple protective effects		Functional role has been suggested	Both direct and indirect effects (modification in the synthesis of lipids, reduction of inflammation)	[24,30]
Pancreas	Augments the action of glucose. Increases secretion of insulin and somatostatin, decreases glucagon. Proliferative and antiapoptotic effect on the pancreatic β cell	Increases glucagon secretion	Augments the action of glucose. Increases secretion of insulin, somatostatin and glucagon		[31,32,33]
Stomach	Gastric emptying and gastric acid secretion decreased				[34]
Gut	slows transit	Enhances intestinal growth and crypt cell proliferation			[35,36]
Adipose tissue	In vitro studies suggest GLP-1 influences adipogenesis, lipogenesis, and lipolysis		In the presence of insulin, stimulates lipoprotein lipase (by increasing gene transcription), adipogenesis. increases weight gain, increases glucose uptake, preadipocyte differentiation.	Based on pre-clinical studies and cultured cell lines. Protein kinase B/ LKB1protein kinase/AMP activated protein kinase signalling module is involved in these processes.	[37,38,39,40,41]
Kidneys	Part of the gut-renal axis. Adjusts excretion of nutrients and electrolytes according to intake		No major effect known		[42]
Bones	Influences bone turnover and resorption		Influences bone turnover and resorption	Possible mechanism is to facilitate optimal use of absorbed nutrients.	[43]

**Table 2:** Hormonal functions in maintaining energy homeostasis.

Hormone	Source and Structure	Function	References
Ghrelin	Gastric-derived 28 amino acid acylated peptide, a ligand of the growth hormone secratogogue receptor type 1a (GHS-R1a), which stimulates GH secretion	Stimulates food intake and increases adiposity	[94,95]
Secretin	Proximal duodenum	Satiation signal?	[98]
Gastrin	Secreted by the G cells in the antral and duodenal mucosa	Stimulates acid secretion and growth of the fundic mucosa	[134]
Cholecystokinin	Small intestine	Satiation signal	[129]
PYY	L cells of the gut	Satiation signal	[109]
Glicentin	Glicentin belongs to the pro-glucagon-derived peptides. It is thought to be mainly synthesised by the L-cells in the ileum	Possible role in intestinal trophicity and motility, gastric acid secretion, and insulin-releasing action. Further studies are required for a better understanding of function	[135]
Oxyntomodulin	Oxyntomodulin is a 37 aa polypeptide formed from proglucagon and synthesised in the L-cells of the gut	Agonist of the glucagon receptor and GLP1R but with lower receptor affinity. In mice may transiently decrease food intake. Can cause weight loss in humans and rodents	[136,137,138,139]
Pancreatic Polypeptide	Pancreas	Satiation signal	[17]
Neurotensin	Expressed in the brain and GIT. Binding mediated by neurotensin receptor 1 and neurotensin receptor 2, both G-protein coupled receptors.	Brain neurotensin has anorectic activity. It has been suggested that neurotensin plays a role in intestinal fat absorption	[140,141]
Amylin	Co-secreted with insulin by pancreatic β cells. Amylin is centrally expressed in several neurons in the brain. Amylin acts through the AMY 1–3 receptors. AMY is a heterodimer of the calcitonin receptor core, which is a G-protein-coupled receptor. Area postrema has a high density of AMY and is directly targeted by amylin. AMY is widely distributed throughout the brain.	Satiating hormone, reduces eating by a meal size effect and slows gastric emptying. Reduces glucagon secretion. Rodent studies suggest that amylin (i) has a sensitising effect towards leptin (ii) and may have an axonal development role in the area postrema and hypothalamus.	[142,143,144]
NPY	NPY is a 36 amino acid neuropeptide found in the central and peripheral nervous system	Orexigenic neuropeptide	[117]
FGF19	Secreted in the distal ileum. Through its intestinal receptor farnesoid X receptor (FXR) bile acids can induce FGF19	Studies in transgenic mice suggest that FGF19 has a role in increased energy expenditure	[145,146]
Orexin	Produced in the hypothalamus, orexin A and B. Receptors are orexin receptors 1 and 2 (OX1R and OX2R)	In normal rats and mice, central administration of orexin increases food intake, blood pressure, and sympathetic nerve activity. Rodent studies suggest that OX1R is involved in obesity and OX2R in thermogenesis, playing a role in energy balance. It is suggested that orexin modulates serotonin’s involvement in energy balance.	[147,148]
Obestatin	Encoded by the ghrelin gene, is widely distributed, and is widespread in the GIT tract	A variety of effects have been proposed, including cell survival and proliferation. In vitro and rodent studies suggest obestatin involvement in (i) adipocyte metabolism and (ii) insulin, somatostatin, PP, and glucagon secretion in the pancreas	[149]
Serotonin	Neurotransmitter. CCK and GLP-1 stimulate serotoninergic neurons	Central serotonin is anorexigenic and stimulates brown adipose tissue, peripheral serotonin promotes energy absorption and storage.	[150]
Resistin	Produced mainly by monocytes/macrophages and in small amounts by adipocytes.	Increased in obesity and thought to contribute to insulin resistance	[151]
GDF15	Growth Differentation factor 15 a stress hormone secreted by cells in the body.	Rodent studies suggest that GDF15 can reduce food intake. GDF15 is thought to play a role in weight loss associated with sickness	[152]

**Table 3:** Summary of selected changes following metabolic bariatric surgery.

GIT Hormones	Adipokines
Ghrelin (D)	Adiponectin (I)
GLP-1 (I)	Leptin (D)
GLP(2) (I)	
PYY (I)	
CCK (I)	
Oxyntomodulin	
**Other factors**	
Bile Acids (I)	

I: increase; D: decrease.

## Data Availability

The data presented in this study are available in the references cited and also on request from the corresponding author.

## References

[1] Gribble F.M., Reimann F. (2019). Function and mechanisms of enteroendocrine cells and gut hormones in metabolism. Nat. Rev. Endocrinol..

[2] Park H.-K., Ahima R.S. (2015). Physiology of leptin: Energy homeostasis, neuroendocrine function and metabolism. Metabolism.

[3] Vohra M.S., Benchoula K., Serpell C.J., Hwa W.E. (2022). AgRP/NPY and POMC neurons in the arcuate nucleus and their potential role in treatment of obesity. Eur. J. Pharmacol..

[4] Ward Z.J., Bleich S.N., Cradock A.L., Barrett J.L., Giles C.M., Flax C., Long M.W., Gortmaker S.L. (2019). Projected U.S. state-level prevalence of adult obesity and severe obesity. N. Engl. J. Med..

[5] Ruban A., Stoenchev K., Ashrafian H., Teare J. (2019). Current treatments for obesity. Clin. Med..

[6] Sohn J.-W. (2015). Network of hypothalamic neurons that control appetite. BMB Rep..

[7] Zhan C., Zhou J., Feng Q., Zhang J.-E., Lin S., Bao J., Wu P., Luo M. (2013). Acute and Long-Term Suppression of Feeding Behavior by POMC Neurons in the Brainstem and Hypothalamus, Respectively. J. Neurosci..

[8] Mountjoy K.G. (2015). Pro-Opiomelanocortin (POMC) Neurones, POMC-Derived Peptides, Melanocortin Receptors and Obesity: How Understanding of this System has Changed Over the Last Decade. J. Neuroendocr..

[9] Qiu J., Zhang C., Borgquist A., Nestor C.C., Smith A.W., Bosch M.A., Ku S., Wagner E.J., Rønnekleiv O.K., Kelly M.J. (2014). Insulin Excites Anorexigenic Proopiomelanocortin Neurons via Activation of Canonical Transient Receptor Potential Channels. Cell Metab..

[10] Quarta C., Claret M., Zeltser L.M., Williams K.W., Yeo G.S.H., Tschöp M.H., Diano S., Brüning J.C., Cota D. (2021). POMC neuronal heterogeneity in energy balance and beyond: An integrated view. Nat. Metab..

[11] Yoon N.A., Diano S. (2021). Hypothalamic glucose-sensing mechanisms. Diabetologia.

[12] Sohn Y.B. (2022). Genetic obesity: An update with emerging therapeutic approaches. Ann. Pediatr. Endocrinol. Metab..

[13] Baldini G., Phelan K.D. (2019). The melanocortin pathway and control of appetite-progress and therapeutic implications. J. Endocrinol..

[14] Denis R.G., Joly-Amado A., Webber E., Langlet F., Schaeffer M., Padilla S.L., Cansell C., Dehouck B., Castel J., Delbès A.-S. (2015). Palatability Can Drive Feeding Independent of AgRP Neurons. Cell Metab..

[15] Benite-Ribeiro S.A., Putt D.A., Soares-Filho M.C., Santos J.M. (2016). The link between hypothalamic epigenetic modifications and long-term feeding control. Appetite.

[16] Schwartz M.W., Woods S.C., Porte D., Seeley R.J., Baskin D.G. (2000). Central nervous system control of food intake. Nature.

[17] Khandekar N., Berning B.A., Sainsbury A., Lin S. (2015). The role of pancreatic polypeptide in the regulation of energy homeostasis. Mol. Cell. Endocrinol..

[18] Pais R., Gribble F.M., Reimann F. (2016). Stimulation of incretin secreting cells. Ther. Adv. Endocrinol. Metab..

[19] El K., Campbell J.E. (2020). The role of GIP in α-cells and glucagon secretion. Peptides.

[20] McLean M.A., Wong C.K., Campbell J.E., Hodson D.J., Trapp S., Drucker D.J. (2021). Revisiting the Complexity of GLP-1 Action from Sites of Synthesis to Receptor Activation. Endocr. Rev..

[21] Chaudhri O., Small C., Bloom S. (2006). Gastrointestinal hormones regulating appetite. Philos. Trans. R. Soc. B Biol. Sci..

[22] Nauck M.A., Quast D.R., Wefers J., Pfeiffer A.F.H. (2021). The evolving story of incretins (GIP and GLP-1) in metabolic and cardiovascular disease: A pathophysiological update. Diabetes Obes. Metab..

[23] El-Jamal N., Erdual E., Neunlist M., Koriche D., Dubuquoy C., Maggiotto F., Chevalier J., Berrebi D., Dubuquoy L., Boulanger E. (2014). Glugacon-like peptide-2: Broad receptor expression, limited therapeutic effect on intestinal inflammation and novel role in liver regeneration. Am. J. Physiol. Liver Physiol..

[24] Zhao X., Wang M., Wen Z., Lu Z., Cui L., Fu C., Xue H., Liu Y., Zhang Y. (2021). GLP-1 Receptor Agonists: Beyond Their Pancreatic Effects. Front. Endocrinol..

[25] Flint A., Raben A., Ersbøll A., Holst J., Astrup A. (2001). The effect of physiological levels of glucagon-like peptide-1 on appetite, gastric emptying, energy and substrate metabolism in obesity. Int. J. Obes..

[26] Heppner K.M., Kirigiti M., Secher A., Paulsen S.J., Buckingham R., Pyke C., Knudsen L.B., Vrang N., Grove K.L. (2015). Expression and Distribution of Glucagon-Like Peptide-1 Receptor mRNA, Protein and Binding in the Male Nonhuman Primate (*Macaca mulatta*) Brain. Endocrinology.

[27] Vrang N., Larsen P.J. (2010). Preproglucagon derived peptides GLP-1, GLP-2 and oxyntomodulin in the CNS: Role of peripherally secreted and centrally produced peptides. Prog. Neurobiol..

[28] Adriaenssens A., Gribble F., Reimann F. (2020). The glucose-dependent insulinotropic polypeptide signaling axis in the central nervous system. Peptides.

[29] Vrang N., Hansen M., Larsen P.J., Tang-Christensen M. (2007). Characterization of brainstem preproglucagon projections to the paraventricular and dorsomedial hypothalamic nuclei. Brain Res..

[30] Heimbürger S.M., Bergmann N.C., Augustin R., Gasbjerg L.S., Christensen M.B., Knop F.K. (2020). Glucose-dependent insulinotropic polypeptide (GIP) and cardiovascular disease. Peptides.

[31] Nauck M.A., Heimesaat M.M., Behle K., Holst J.J., Nauck M.S., Ritzel R., Hüfner M., Schmiegel W.H. (2002). Effects of Glucagon-Like Peptide 1 on Counterregulatory Hormone Responses, Cognitive Functions, and Insulin Secretion during Hyperinsulinemic, Stepped Hypoglycemic Clamp Experiments in Healthy Volunteers. J. Clin. Endocrinol. Metab..

[32] Brubaker P.L., Drucker D.J. (2004). Minireview: Glucagon-Like Peptides Regulate Cell Proliferation and Apoptosis in the Pancreas, Gut, and Central Nervous System. Endocrinology.

[33] Meier J.J., Nauck M.A., Pott A., Heinze K., Goetze O., Bulut K., Schmidt W.E., Gallwitz B., Holst J.J. (2006). Glucagon-Like Peptide 2 Stimulates Glucagon Secretion, Enhances Lipid Absorption, and Inhibits Gastric Acid Secretion in Humans. Gastroenterology.

[34] Meier J.J., Goetze O., Anstipp J., Hagemann D., Holst J.J., Schmidt W.E., Gallwitz B., Nauck M.A. (2004). Gastric inhibitory polypeptide does not inhibit gastric emptying in humans. Am. J. Physiol. Endocrinol. Metab..

[35] Hellström P.M., Näslund E., Edholm T., Schmidt P.T., Kristensen J., Theodorsson E., Holst J.J., Efendic S. (2008). GLP-1 suppresses gastrointestinal motility and inhibits the migrating motor complex in healthy subjects and patients with irritable bowel syndrome. Neurogastroenterol. Motil..

[36] Brubaker P.L. (2018). Glucagon-like Peptide-2 and the Regulation of Intestinal Growth and Function. Compr. Physiol..

[37] Miyawaki K., Yamada Y., Ban N., Ihara Y., Tsukiyama K., Zhou H., Fujimoto S., Oku A., Tsuda K., Toyokuni S. (2002). Inhibition of gastric inhibitory polypeptide signaling prevents obesity. Nat. Med..

[38] Kim S.-J., Nian C., McIntosh C.H.S. (2007). Activation of Lipoprotein Lipase by Glucose-dependent Insulinotropic Polypeptide in Adipocytes. J. Biol. Chem..

[39] Kim S.-J., Nian C., McIntosh C.H. (2010). GIP increases human adipocyte LPL expression through CREB and TORC2-mediated trans-activation of the LPL gene. J. Lipid Res..

[40] McIntosh C.H., Widenmaier S., Kim S. (2012). Glucose-dependent insulinotropic polypeptide signaling in pancreatic β-cells and adipocytes. J. Diabetes Investig..

[41] El Bekay R., Coín-Aragüez L., Fernández-García D., Oliva-Olivera W., Bernal-López R., Clemente-Postigo M., Delgado-Lista J., Diaz-Ruiz A., Guzman-Ruiz R., Vázquez-Martínez R. (2016). Effects of glucagon-like peptide-1 on the differentiation and metabolism of human adipocytes. Br. J. Pharmacol..

[42] Muskiet M.H.A., Tonneijck L., Smits M.M., Van Baar M.J.B., Kramer M.H.H., Hoorn E.J., Joles J.A., Van Raalte D.H. (2017). GLP-1 and the kidney: From physiology to pharmacology and outcomes in diabetes. Nat. Rev. Nephrol..

[43] Sherk V.D., Schauer I., Shah V.N. (2020). Update on the Acute Effects of Glucose, Insulin, and Incretins on Bone Turnover In Vivo. Curr. Osteoporos. Rep..

[44] Seino Y., Fukushima M., Yabe D. (2010). GIP and GLP-1, the two incretin hormones: Similarities and differences. J. Diabetes Investig..

[45] Rouillé Y., Martin S., Steiner D.F. (1995). Differential Processing of Proglucagon by the Subtilisin-like Prohormone Convertases PC2 and PC3 to Generate either Glucagon or Glucagon-like Peptide. J. Biol. Chem..

[46] Friis-Hansen L., Lacourse K., Samuelson L., Holst J. (2001). Attenuated processing of proglucagon and glucagon-like peptide-1 in carboxypeptidase E-deficient mice. J. Endocrinol..

[47] Ørskov C., Rabenhøj L., Wettergren A., Kofod H., Holst J.J. (1994). Tissue and Plasma Concentrations of Amidated and Glycine-Extended Glucagon-Like Peptide I in Humans. Diabetes.

[48] Brubaker P.L., Crivici A., Izzo A., Ehrlich P., Tsai C.-H., Drucker D.J. (1997). Circulating and Tissue Forms of the Intestinal Growth Factor, Glucagon-Like Peptide-2*. Endocrinology.

[49] Takeda J., Seino Y., Tanaka K., Fukumoto H., Kayano T., Takahashi H., Mitani T., Kurono M., Suzuki T., Tobe T. (1987). Sequence of an intestinal cDNA encoding human gastric inhibitory polypeptide precursor. Proc. Natl. Acad. Sci. USA.

[50] Kieffer T.J., McIntosh C.H., Pederson R.A. (1995). Degradation of glucose-dependent insulinotropic polypeptide and truncated glucagon-like peptide 1 in vitro and in vivo by dipeptidyl peptidase IV. Endocrinology.

[51] Mentlein R. (2009). Mechanisms underlying the rapid degradation and elimination of the incretin hormones GLP-1 and GIP. Best Pract. Res. Clin. Endocrinol. Metab..

[52] Meier J.J., Nauck M.A., Kranz D., Holst J.J., Deacon C.F., Gaeckler D., Schmidt W.E., Gallwitz B. (2004). Secretion, Degradation, and Elimination of Glucagon-Like Peptide 1 and Gastric Inhibitory Polypeptide in Patients with Chronic Renal Insufficiency and Healthy Control Subjects. Diabetes.

[53] Hartmann B., Harr M.B., Jeppesen P.B., Wojdemann M., Deacon C.F., Mortensen P.B., Holst J.J. (2000). In Vivo and in Vitro Degradation of Glucagon-Like Peptide-2 in Humans1. J. Clin. Endocrinol. Metab..

[54] Gasbjerg L.S., Helsted M.M., Hartmann B., Jensen M.H., Gabe M.B.N., Sparre-Ulrich A.H., Veedfald S., Stensen S., Lanng A.R., Bergmann N.C. (2019). Separate and Combined Glucometabolic Effects of Endogenous Glucose-Dependent Insulinotropic Polypeptide and Glucagon-like Peptide 1 in Healthy Individuals. Diabetes.

[55] Nauck M.A., Meier J.J. (2019). GIP and GLP-1: Stepsiblings Rather Than Monozygotic Twins Within the Incretin Family. Diabetes.

[56] Gribble F.M., Williams L., Simpson A.K., Reimann F. (2003). A Novel Glucose-Sensing Mechanism Contributing to Glucagon-Like Peptide-1 Secretion from the GLUTag Cell Line. Diabetes.

[57] Röder P.V., Geillinger K.E., Zietek T.S., Thorens B., Koepsell H., Daniel H. (2014). The Role of SGLT1 and GLUT2 in Intestinal Glucose Transport and Sensing. PLoS ONE.

[58] Pais R., Gribble F.M., Reimann F. (2016). Signalling pathways involved in the detection of peptones by murine small intestinal enteroendocrine L-cells. Peptides.

[59] Batterham R.L., Heffron H., Kapoor S., Chivers J.E., Chandarana K., Herzog H., Le Roux C.W., Thomas E.L., Bell J.D., Withers D.J. (2006). Critical role for peptide YY in protein-mediated satiation and body-weight regulation. Cell Metab..

[60] Feltrin K.L., Little T.J., Meyer J.H., Horowitz M., Smout A.J.P.M., Wishart J., Pilichiewicz A.N., Rades T., Chapman I.M., Feinle-Bisset C. (2004). Effects of intraduodenal fatty acids on appetite, antropyloroduodenal motility, and plasma CCK and GLP-1 in humans vary with their chain length. Am. J. Physiol. Regul. Integr. Comp. Physiol..

[61] Feltrin K.L., Little T.J., Meyer J.H., Horowitz M., Rades T., Wishart J., Feinle-Bisset C. (2007). Effects of lauric acid on upper gut motility, plasma cholecystokinin and peptide YY, and energy intake are load, but not concentration, dependent in humans. J. Physiol..

[62] Briscoe C.P., Tadayyon M., Andrews J.L., Benson W.G., Chambers J.K., Eilert M.M., Ellis C., Elshourbagy N.A., Goetz A.S., Minnick D.T. (2003). The Orphan G Protein-coupled Receptor GPR40 Is Activated by Medium and Long Chain Fatty Acids. J. Biol. Chem..

[63] Ridner G., Bartoov-Shifman R., Zalogin T., Avnit-Sagi T., Bahar K., Sharivkin R., Kantorovich L., Weiss S., Walker M.D. (2008). Regulation of the *GPR40* locus: Towards a molecular understanding. Biochem. Soc. Trans..

[64] Iwasaki K., Harada N., Sasaki K., Yamane S., Iida K., Suzuki K., Hamasaki A., Nasteska D., Shibue K., Joo E. (2015). Free Fatty Acid Receptor GPR120 Is Highly Expressed in Enteroendocrine K Cells of the Upper Small Intestine and Has a Critical Role in GIP Secretion After Fat Ingestion. Endocrinology.

[65] Sankoda A., Harada N., Kato T., Ikeguchi E., Iwasaki K., Yamane S., Murata Y., Hirasawa A., Inagaki N. (2019). Free fatty acid receptors, G protein-coupled receptor 120 and G protein-coupled receptor 40, are essential for oil-induced gastric inhibitory polypeptide secretion. J. Diabetes Investig..

[66] Overton H.A., Babbs A.J., Doel S.M., Fyfe M.C., Gardner L.S., Griffin G., Jackson H.C., Procter M.J., Rasamison C.M., Tang-Christensen M. (2006). Deorphanization of a G protein-coupled receptor for oleoylethanolamide and its use in the discovery of small-molecule hypophagic agents. Cell Metab..

[67] Edfalk S., Steneberg P., Edlund H. (2008). *Gpr40* Is Expressed in Enteroendocrine Cells and Mediates Free Fatty Acid Stimulation of Incretin Secretion. Diabetes.

[68] Hirasawa A., Tsumaya K., Awaji T., Katsuma S., Adachi T., Yamada M., Sugimoto Y., Miyazaki S., Tsujimoto G. (2005). Free fatty acids regulate gut incretin glucagon-like peptide-1 secretion through GPR120. Nat. Med..

[69] Chu Z.-L., Carroll C., Alfonso J., Gutierrez V., He H., Lucman A., Pedraza M., Mondala H., Gao H., Bagnol D. (2008). A Role for Intestinal Endocrine Cell-Expressed G Protein-Coupled Receptor 119 in Glycemic Control by Enhancing Glucagon-Like Peptide-1 and Glucose-Dependent Insulinotropic Peptide Release. Endocrinology.

[70] Tolhurst G., Heffron H., Lam Y.S., Parker H.E., Habib A.M., Diakogiannaki E., Cameron J., Grosse J., Reimann F., Gribble F.M. (2012). Short-Chain Fatty Acids Stimulate Glucagon-Like Peptide-1 Secretion via the G-Protein-Coupled Receptor FFAR2. Diabetes.

[71] Tremaroli V., Bäckhed F. (2012). Functional interactions between the gut microbiota and host metabolism. Nature.

[72] Wichmann A., Allahyar A., Greiner T.U., Plovier H., Lundén G., Larsson T., Drucker D.J., Delzenne N.M., Cani P.D., Bäckhed F. (2013). Microbial Modulation of Energy Availability in the Colon Regulates Intestinal Transit. Cell Host Microbe.

[73] Katsuma S., Hirasawa A., Tsujimoto G. (2005). Bile acids promote glucagon-like peptide-1 secretion through TGR5 in a murine enteroendocrine cell line STC-1. Biochem. Biophys. Res. Commun..

[74] Ullmer C., Sanchez R.A., Sprecher U., Raab S., Mattei P., Dehmlow H., Sewing S., Iglesias A., Beauchamp J., Conde-Knape K. (2013). Systemic bile acid sensing by G protein-coupled bile acid receptor 1 (GPBAR1) promotes PYY and GLP-1 release. Br. J. Pharmacol..

[75] Hylemon P.B., Zhou H., Pandak W.M., Ren S., Gil G., Dent P. (2009). Bile acids as regulatory molecules. J. Lipid Res..

[76] Parker H., Wallis K., le Roux C., Wong K., Reimann F., Gribble F. (2012). Molecular mechanisms underlying bile acid-stimulated glucagon-like peptide-1 secretion. Br. J. Pharmacol..

[77] Thomas C., Gioiello A., Noriega L., Strehle A., Oury J., Rizzo G., Macchiarulo A., Yamamoto H., Mataki C., Pruzanski M. (2009). TGR5-Mediated Bile Acid Sensing Controls Glucose Homeostasis. Cell Metab..

[78] Brighton C.A., Rievaj J., Kuhre R.E., Glass L.L., Schoonjans K., Holst J.J., Gribble F.M., Reimann F. (2015). Bile Acids Trigger GLP-1 Release Predominantly by Accessing Basolaterally Located G Protein–Coupled Bile Acid Receptors. Endocrinology.

[79] Gagnon J., Sauvé M., Zhao W., Stacey H.M., Wiber S.C., Bolz S.-S., Brubaker P.L. (2015). Chronic Exposure to TNFα Impairs Secretion of Glucagon-Like Peptide-1. Endocrinology.

[80] Allen T.L., Whitham M., Febbraio M.A. (2012). IL-6 Muscles in on the Gut and Pancreas to Enhance Insulin Secretion. Cell Metab..

[81] Ellingsgaard H., Hauselmann I., Schuler B., Habib A.M., Baggio L.L., Meier D.T., Eppler E., Bouzakri K., Wueest S., Muller Y.D. (2011). Interleukin-6 enhances insulin secretion by increasing glucagon-like peptide-1 secretion from L cells and alpha cells. Nat. Med..

[82] Pais R., Zietek T., Hauner H., Daniel H., Skurk T. (2014). RANTES (CCL5) reduces glucose-dependent secretion of glucagon-like peptides 1 and 2 and impairs glucose-induced insulin secretion in mice. Am. J. Physiol. Liver Physiol..

[83] Flock G.B., Cao X., Maziarz M., Drucker D.J. (2012). Activation of Enteroendocrine Membrane Progesterone Receptors Promotes Incretin Secretion and Improves Glucose Tolerance in Mice. Diabetes.

[84] Lim G.E., Huang G.J., Flora N., LeRoith D., Rhodes C.J., Brubaker P.L. (2009). Insulin Regulates Glucagon-Like Peptide-1 Secretion from the Enteroendocrine L Cell. Endocrinology.

[85] Kappe C., Fransson L., Wolbert P., Ortsäter H. (2015). Glucocorticoids suppress GLP-1 secretion: Possible contribution to their diabetogenic effects. Clin. Sci..

[86] Rahman S., Hossain K.S., Das S., Kundu S., Adegoke E.O., Rahman A., Hannan A., Uddin J., Pang M.-G. (2021). Role of Insulin in Health and Disease: An Update. Int. J. Mol. Sci..

[87] Mitchell C.S., Begg D.P. (2021). The regulation of food intake by insulin in the central nervous system. J. Neuroendocr..

[88] Janah L., Kjeldsen S., Galsgaard K.D., Winther-Sørensen M., Stojanovska E., Pedersen J., Knop F.K., Holst J.J., Albrechtsen N.J.W. (2019). Glucagon Receptor Signaling and Glucagon Resistance. Int. J. Mol. Sci..

[89] Woods S.C., Lutz T.A., Geary N., Langhans W. (2006). Pancreatic signals controlling food intake; insulin, glucagon and amylin. Philos. Trans. R. Soc. B Biol. Sci..

[90] Kleinert M., Sachs S., Habegger K.M., Hofmann S.M., Müller T.D. (2019). Glucagon Regulation of Energy Expenditure. Int. J. Mol. Sci..

[91] Aranda-Domene R., Orenes-Piñero E., Arribas-Leal J.M., Canovas-Lopez S., Hernández-Cascales J. (2023). Evidence for a lack of inotropic and chronotropic effects of glucagon and glucagon receptors in the human heart. Cardiovasc. Diabetol..

[92] Parmley W.W., Glick G., Sonnenblick E.H. (1968). Cardiovascular Effects of Glucagon in Man. N. Engl. J. Med..

[93] Cummings D.E., Purnell J.Q., Frayo R.S., Schmidova K., Wisse B.E., Weigle D.S. (2001). A Preprandial Rise in Plasma Ghrelin Levels Suggests a Role in Meal Initiation in Humans. Diabetes.

[94] Yanagi S., Sato T., Kangawa K., Nakazato M. (2018). The Homeostatic Force of Ghrelin. Cell Metab..

[95] Mani B.K., Zigman J.M. (2017). Ghrelin as a Survival Hormone. Trends Endocrinol. Metab..

[96] Deschaine S.L., Leggio L. (2022). From “Hunger Hormone” to “It’s Complicated”: Ghrelin Beyond Feeding Control. Physiology.

[97] Sekar R., Chow B.K.C. (2014). Lipolytic actions of secretin in mouse adipocytes. J. Lipid Res..

[98] Schnabl K., Li Y., U-Din M., Klingenspor M. (2021). Secretin as a Satiation Whisperer With the Potential to Turn into an Obesity-curbing Knight. Endocrinology.

[99] Cheng C.Y.Y., Chu J.Y.S., Chow B.K.C. (2011). Central and Peripheral Administration of Secretin Inhibits Food Intake in Mice through the Activation of the Melanocortin System. Neuropsychopharmacology.

[100] Laurila S., Sun L., Lahesmaa M., Schnabl K., Laitinen K., Klén R., Li Y., Balaz M., Wolfrum C., Steiger K. (2021). Secretin activates brown fat and induces satiation. Nat. Metab..

[101] Li Y., Schnabl K., Gabler S.-M., Willershäuser M., Reber J., Karlas A., Laurila S., Lahesmaa M., Din M.U., Bast-Habersbrunner A. (2018). Secretin-Activated Brown Fat Mediates Prandial Thermogenesis to Induce Satiation. Cell.

[102] Sekar R., Chow B.K.C. (2014). Secretin receptor-knockout mice are resistant to high-fat diet-induced obesity and exhibit impaired intestinal lipid absorption. FASEB J..

[103] Laurila S., Rebelos E., Lahesmaa M., Sun L., Schnabl K., Peltomaa T.-M., Klén R., U-Din M., Honka M.-J., Eskola O. (2022). Novel effects of the gastrointestinal hormone secretin on cardiac metabolism and renal function. Am. J. Physiol. Endocrinol. Metab..

[104] Gustafson E.L., E Smith K., Durkin M.M., Walker M.W., Gerald C., Weinshank R., A Branchek T. (1997). Distribution of the neuropeptide Y Y2 receptor mRNA in rat central nervous system. Mol. Brain Res..

[105] Zhang L., Macia L., Turner N., Enriquez R.F., Riepler S.J., Nguyen A.D., Lin S., Lee N.J., Shi Y.C., Yulyaningsih E. (2010). Peripheral neuropeptide Y Y1 receptors regulate lipid oxidation and fat accretion. Int. J. Obes..

[106] Shi Y., Lin S., Castillo L., Aljanova A., Enriquez R.F., Nguyen A.D., Baldock P.A., Zhang L., Bijker M.S., Macia L. (2011). Peripheral-Specific Y2 Receptor Knockdown Protects Mice from High-Fat Diet-Induced Obesity. Obesity.

[107] Adrian T., Ferri G.-L., Bacarese-Hamilton A., Fuessl H., Polak J., Bloom S. (1985). Human distribution and release of a putative new gut hormone, peptide YY. Gastroenterology.

[108] Batterham R.L., Cohen M.A., Ellis S.M., Le Roux C.W., Withers D.J., Frost G.S., Ghatei M.A., Bloom S.R. (2003). Inhibition of Food Intake in Obese Subjects by Peptide YY3–36. N. Engl. J. Med..

[109] Batterham R.L., Bloom S.R. (2003). The Gut Hormone Peptide YY Regulates Appetite. Ann. N. Y. Acad. Sci..

[110] Koda S., Date Y., Murakami N., Shimbara T., Hanada T., Toshinai K., Niijima A., Furuya M., Inomata N., Osuye K. (2005). The Role of the Vagal Nerve in Peripheral PYY3–36-Induced Feeding Reduction in Rats. Endocrinology.

[111] Adrian T.E., Bloom S.R., Bryant M.G., Polak J.M., Heitz P.H., Barnes A.J. (1976). Distribution and release of human pancreatic polypeptide. Gut.

[112] Blomqvist A.G., Herzog H. (1997). Y-receptor subtypes—How many more?. Trends Neurosci..

[113] Asakawa A., Inui A., Yuzuriha H., Ueno N., Katsuura G., Fujimiya M., Fujino M.A., Niijima A., Meguid M.M., Kasuga M. (2003). Characterization of the effects of pancreatic polypeptide in the regulation of energy balance. Gastroenterology.

[114] Lin S., Shi Y.-C., Yulyaningsih E., Aljanova A., Zhang L., Macia L., Nguyen A.D., Lin E.-J.D., During M.J., Herzog H. (2009). Critical Role of Arcuate Y4 Receptors and the Melanocortin System in Pancreatic Polypeptide-Induced Reduction in Food Intake in Mice. PLoS ONE.

[115] Sainsbury A., Shi Y.-C., Zhang L., Aljanova A., Lin Z., Nguyen A.D., Herzog H., Lin S. (2010). Y4 receptors and pancreatic polypeptide regulate food intake via hypothalamic orexin and brain-derived neurotropic factor dependent pathways. Neuropeptides.

[116] Yan C., Zeng T., Lee K., Nobis M., Loh K., Gou L., Xia Z., Gao Z., Bensellam M., Hughes W. (2021). Peripheral-specific Y1 receptor antagonism increases thermogenesis and protects against diet-induced obesity. Nat. Commun..

[117] Loh K., Herzog H., Shi Y.-C. (2015). Regulation of energy homeostasis by the NPY system. Trends Endocrinol. Metab..

[118] Fetissov S.O., Kopp J., Hökfelt T. (2004). Distribution of NPY receptors in the hypothalamus. Neuropeptides.

[119] Bi S., Li L. (2013). Browning of white adipose tissue: Role of hypothalamic signaling. Ann. N. Y. Acad. Sci..

[120] Bohórquez D.V., Samsa L.A., Roholt A., Medicetty S., Chandra R., Liddle R.A. (2014). An Enteroendocrine Cell—Enteric Glia Connection Revealed by 3D Electron Microscopy. PLoS ONE.

[121] Rehfeld J.F. (2017). Cholecystokinin—From Local Gut Hormone to Ubiquitous Messenger. Front. Endocrinol..

[122] Rehfeld J.F., Sun G., Christensen T., Hillingsø J.G. (2001). The Predominant Cholecystokinin in Human Plasma and Intestine Is Cholecystokinin-33^1^. J. Clin. Endocrinol. Metab..

[123] Liddle R.A., Goldfine I.D., Rosen M.S., Taplitz R.A., Williams J.A. (1985). Cholecystokinin bioactivity in human plasma. Molecular forms, responses to feeding, and relationship to gallbladder contraction. J. Clin. Investig..

[124] Hoffmann P., Eberlein G.A., Reeve J.R., Bünte R.H., Grandt D., Goebell H., Eysselein V.E. (1993). Comparison of clearance and metabolism of infused cholecystokinins 8 and 58 in dogs. Gastroenterology.

[125] Dodd J., Kelly J. (1981). The actions of cholecystokinin and related peptides on pyramidal neurones of the mammalian hippocampus. Brain Res..

[126] Gallmann E., Arsenijevic D., Spengler M., Williams G., Langhans W. (2005). Effect of CCK-8 on insulin-induced hyperphagia and hypothalamic orexigenic neuropeptide expression in the rat. Peptides.

[127] Kobelt P., Tebbe J.J., Tjandra I., Stengel A., Bae H.-G., Andresen V., van der Voort I.R., Veh R.W., Werner C.R., Klapp B.F. (2005). CCK inhibits the orexigenic effect of peripheral ghrelin. Am. J. Physiol. Integr. Comp. Physiol..

[128] De Lartigue G., Dimaline R., Varro A., Raybould H., De La Serre C.B., Dockray G.J. (2010). Cocaine- and Amphetamine-Regulated Transcript Mediates the Actions of Cholecystokinin on Rat Vagal Afferent Neurons. Gastroenterology.

[129] Chandra R., Liddle R.A. (2007). Cholecystokinin. Curr. Opin. Endocrinol. Diabetes.

[130] Rorsman P., Huising M.O. (2018). The somatostatin-secreting pancreatic δ-cell in health and disease. Nat. Rev. Endocrinol..

[131] Shamsi B.H., Chatoo M., Xu X.K., Xu X., Chen X.Q. (2021). Versatile Functions of Somatostatin and Somatostatin Receptors in the Gastrointestinal System. Front. Endocrinol..

[132] Schubert M.L., Rehfeld J.F. (2019). Gastric Peptides-Gastrin and Somatostatin. Compr. Physiol..

[133] Stengel A., Taché Y. (2019). Central somatostatin signaling and regulation of food intake. Ann. N. Y. Acad. Sci..

[134] Rehfeld J.F. (2021). Gastrin and the Moderate Hypergastrinemias. Int. J. Mol. Sci..

[135] Raffort J., Lareyre F., Massalou D., Fénichel P., Panaïa-Ferrari P., Chinetti G. (2017). Insights on glicentin, a promising peptide of the proglucagon family. Biochem. Medica.

[136] Holst J.J., Albrechtsen N.J., Gabe M.B.N., Rosenkilde M.M. (2018). Oxyntomodulin: Actions and role in diabetes. Peptides.

[137] Baggio L.L., Huang Q., Brown T.J., Drucker D.J. (2004). Oxyntomodulin and glucagon-like peptide-1 differentially regulate murine food intake and energy expenditure. Gastroenterology.

[138] Kosinski J.R., Hubert J., Carrington P.E., Chicchi G.G., Mu J., Miller C., Cao J., Bianchi E., Pessi A., SinhaRoy R. (2012). The Glucagon Receptor Is Involved in Mediating the Body Weight-Lowering Effects of Oxyntomodulin. Obesity.

[139] Laferrère B., Swerdlow N., Bawa B., Arias S., Bose M., Oliván B., Teixeira J., McGinty J., Rother K.I. (2010). Rise of Oxyntomodulin in Response to Oral Glucose after Gastric Bypass Surgery in Patients with Type 2 Diabetes. J. Clin. Endocrinol. Metab..

[140] Li J., Song J., Zaytseva Y.Y., Liu Y., Rychahou P., Jiang K., Starr M.E., Kim J.T., Harris J.W., Yiannikouris F.B. (2016). An obligatory role for neurotensin in high-fat-diet-induced obesity. Nature.

[141] Schroeder L.E., Leinninger G.M. (2018). Role of central neurotensin in regulating feeding: Implications for the development and treatment of body weight disorders. Biochim. Biophys. Acta Mol. Basis Dis..

[142] Levin B.E., Lutz T.A. (2017). Amylin and Leptin: Co-Regulators of Energy Homeostasis and Neuronal Development. Trends Endocrinol. Metab..

[143] Lutz T.A. (2022). Creating the amylin story. Appetite.

[144] Boyle C.N., Zheng Y., Lutz T.A. (2022). Mediators of Amylin Action in Metabolic Control. J. Clin. Med..

[145] Tomlinson E., Fu L., John L., Hultgren B., Huang X., Renz M., Stephan J.P., Tsai S.P., Powell-Braxton L., French D. (2002). Transgenic Mice Expressing Human Fibroblast Growth Factor-19 Display Increased Metabolic Rate and Decreased Adiposity. Endocrinology.

[146] Wei M., Cao W.-B., Zhao R.-D., Sun D.-P., Liang Y.-Z., Huang Y.-D., Cheng Z.-W., Ouyang J., Yang W.-S., Yu W.-B. (2023). Fibroblast growth factor 15, induced by elevated bile acids, mediates the improvement of hepatic glucose metabolism after sleeve gastrectomy. World J. Gastroenterol..

[147] Mavanji V., Pomonis B., Kotz C.M. (2022). Orexin, serotonin, and energy balance. WIREs Mech. Dis..

[148] Imperatore R., Palomba L., Cristino L. (2017). Role of Orexin-A in Hypertension and Obesity. Curr. Hypertens. Rep..

[149] Trovato L., Gallo D., Settanni F., Gesmundo I., Ghigo E., Granata R. (2014). Obestatin: Is it really doing something?. Front. Horm. Res..

[150] van Galen K.A., ter Horst K.W., Serlie M.J. (2021). Serotonin, food intake, and obesity. Obes. Rev..

[151] Kirichenko T.V., Markina Y.V., Bogatyreva A.I., Tolstik T.V., Varaeva Y.R., Starodubova A.V. (2022). The Role of Adipokines in Inflammatory Mechanisms of Obesity. Int. J. Mol. Sci..

[152] Siddiqui J.A., Pothuraju R., Khan P., Sharma G., Muniyan S., Seshacharyulu P., Jain M., Nasser M.W., Batra S.K. (2021). Pathophysiological role of growth differentiation factor 15 (GDF15) in obesity, cancer, and cachexia. Cytokine Growth Factor Rev..

[153] Navarro-Perez J., Vidal-Puig A., Carobbio S. (2023). Recent developments in adipose tissue-secreted factors and their target organs. Curr. Opin. Genet. Dev..

[154] Funcke J.-B., Scherer P.E. (2019). Beyond adiponectin and leptin: Adipose tissue-derived mediators of inter-organ communication. J. Lipid Res..

[155] Laiglesia L.M., Escoté X., Sáinz N., Felix-Soriano E., Santamaría E., Collantes M., Fernández-Galilea M., Colón-Mesa I., Martínez-Fernández L., Quesada-López T. (2023). Maresin 1 activates brown adipose tissue and promotes browning of white adipose tissue in mice. Mol. Metab..

[156] Margetic S., Gazzola C., Pegg G.G., Hill R.A. (2002). Leptin: A review of its peripheral actions and interactions. Int. J. Obes. Relat. Metab. Disord..

[157] Moon H.-S., Dalamaga M., Kim S.-Y., Polyzos S.A., Hamnvik O.-P., Magkos F., Paruthi J., Mantzoros C.S. (2013). Leptin’s Role in Lipodystrophic and Nonlipodystrophic Insulin-Resistant and Diabetic Individuals. Endocr. Rev..

[158] Landt M., Lawson G.M., Helgeson J.M., Davila-Roman V.G., Ladenson J.H., Jaffe A.S., Hickner R.C. (1997). Prolonged exercise decreases serum leptin concentrations. Metabolism.

[159] Bouassida A., Zalleg D., Bouassida S., Zaouali M., Feki Y., Zbidi A., Tabka Z. (2006). Leptin, its implication in physical exercise and training: A short review. J. Sports Sci. Med..

[160] Trayhurn P., Duncan J.S., Rayner D.V. (1995). Acute cold-induced suppression of *ob* (obese) gene expression in white adipose tissue of mice: Mediation by the sympathetic system. Biochem. J..

[161] Pereira S., Cline D.L., Glavas M.M., Covey S.D., Kieffer T.J. (2021). Tissue-Specific Effects of Leptin on Glucose and Lipid Metabolism. Endocr. Rev..

[162] Haynes W.G., Morgan D.A., Walsh S.A., Mark A.L., Sivitz W.I. (1997). Receptor-mediated regional sympathetic nerve activation by leptin. J. Clin. Investig..

[163] Pollock B.H., Fischer A.W., Schlein C., Cannon B., Heeren J., Nedergaard J., Côté I., Sakarya Y., Green S.M., Carter C.S. (1997). Leptin increases uncoupling protein expression and energy expenditure. Am. J. Physiol. Metab..

[164] Dodd G.T., Decherf S., Loh K., Simonds S.E., Wiede F., Balland E., Merry T.L., Münzberg H., Zhang Z.-Y., Kahn B.B. (2015). Leptin and Insulin Act on POMC Neurons to Promote the Browning of White Fat. Cell.

[165] Friedman J. (2014). 20 YEARS OF LEPTIN: Leptin at 20: An overview. J. Endocrinol..

[166] Fang H., Judd R.L. (2018). Adiponectin Regulation and Function. Compr. Physiol..

[167] Berg A.H., Combs T.P., Du X., Brownlee M., Scherer P.E. (2001). The adipocyte-secreted protein Acrp30 enhances hepatic insulin action. Nat. Med..

[168] Tomas E., Tsao T.-S., Saha A.K., Murrey H.E., Zhang C.C., Itani S.I., Lodish H.F., Ruderman N.B. (2002). Enhanced muscle fat oxidation and glucose transport by ACRP30 globular domain: Acetyl–CoA carboxylase inhibition and AMP-activated protein kinase activation. Proc. Natl. Acad. Sci. USA.

[169] Kim J.-Y., van de Wall E., Laplante M., Azzara A., Trujillo M.E., Hofmann S.M., Schraw T., Durand J.L., Li H., Li G. (2007). Obesity-associated improvements in metabolic profile through expansion of adipose tissue. J. Clin. Investig..

[170] Kharroubi I., Rasschaert J., Eizirik D.L., Cnop M. (2003). Expression of adiponectin receptors in pancreatic β cells. Biochem. Biophys. Res. Commun..

[171] Patané G., Caporarello N., Marchetti P., Parrino C., Sudano D., Marselli L., Vigneri R., Frittitta L. (2013). Adiponectin increases glucose-induced insulin secretion through the activation of lipid oxidation. Acta Diabetol..

[172] Hui X., Gu P., Zhang J., Nie T., Pan Y., Wu D., Feng T., Zhong C., Wang Y., Lam K.S. (2015). Adiponectin Enhances Cold-Induced Browning of Subcutaneous Adipose Tissue via Promoting M2 Macrophage Proliferation. Cell Metab..

[173] Kadowaki T., Yamauchi T., Kubota N. (2008). The physiological and pathophysiological role of adiponectin and adiponectin receptors in the peripheral tissues and CNS. FEBS Lett..

[174] Krude H., Biebermann H., Schnabel D., Tansek M.Z., Theunissen P., Mullis P.E., Grüters A. (2003). Obesity Due to Proopiomelanocortin Deficiency: Three New Cases and Treatment Trials with Thyroid Hormone and ACTH4–10. J. Clin. Endocrinol. Metab..

[175] Blüher S., Shah S., Mantzoros C.S. (2009). Leptin Deficiency: Clinical Implications and Opportunities for Therapeutic Interventions. J. Investig. Med..

[176] Lubrano-Berthelier C., Cavazos M., Dubern B., Shapiro A., LE Stunff C., Zhang S., Picart F., Govaerts C., Froguel P., Bougnères P. (2003). Molecular Genetics of Human Obesity-Associated MC4R Mutations. Ann. N. Y. Acad. Sci..

[177] Holst B., Schwartz T.W. (2006). Ghrelin receptor mutations—Too little height and too much hunger. J. Clin. Investig..

[178] Folon L., Baron M., Toussaint B., Vaillant E., Boissel M., Scherrer V., Loiselle H., Leloire A., Badreddine A., Balkau B. (2023). Contribution of heterozygous PCSK1 variants to obesity and implications for precision medicine: A case-control study. Lancet Diabetes Endocrinol..

[179] Bray M.S., Boerwinkle E., Hanis C.L. (2000). Sequence Variation within the Neuropeptide Y Gene and Obesity in Mexican Americans. Obes. Res..

[180] Podyma B., Parekh K., Güler A.D., Deppmann C.D. (2021). Metabolic homeostasis via BDNF and its receptors. Trends Endocrinol. Metab..

[181] Singh R.K., Kumar P., Mahalingam K. (2017). Molecular genetics of human obesity: A comprehensive review. Comptes Rendus Biol..

[182] Mahmoud R., Kimonis V., Butler M.G. (2022). Genetics of Obesity in Humans: A Clinical Review. Int. J. Mol. Sci..

[183] Sjöström L., Narbro K., Sjöström C.D., Karason K., Larsson B., Wedel H., Lystig T., Sullivan M., Bouchard C., Carlsson B. (2007). Effects of Bariatric Surgery on Mortality in Swedish Obese Subjects. N. Engl. J. Med..

[184] O’brien P.E., Hindle A., Brennan L., Skinner S., Burton P., Smith A., Crosthwaite G., Brown W. (2019). Long-Term Outcomes After Bariatric Surgery: A Systematic Review and Meta-analysis of Weight Loss at 10 or More Years for All Bariatric Procedures and a Single-Centre Review of 20-Year Outcomes After Adjustable Gastric Banding. Obes. Surg..

[185] Mechanick J.I., Apovian C., Brethauer S., Garvey W.T., Joffe A.M., Kim J., Kushner R.F., Lindquist R., Pessah-Pollack R., Seger J. (2020). Clinical Practice Guidelines for the Perioperative Nutrition, Metabolic, and Nonsurgical Support of Patients Undergoing Bariatric Procedures—2019 Update: Cosponsored by American Association of Clinical Endocrinologists/American College of Endocrinology, The Obesity Society, American Society for Metabolic and Bariatric Surgery, Obesity Medicine Association, and American Society of Anesthesiologists. Obesity.

[186] Eisenberg D., Shikora S.A., Aarts E., Aminian A., Angrisani L., Cohen R.V., de Luca M., Faria S.L., Goodpaster K.P., Haddad A. (2022). 2022 American Society of Metabolic and Bariatric Surgery (ASMBS) and International Federation for the Surgery of Obesity and Metabolic Disorders (IFSO) Indications for Metabolic and Bariatric Surgery. Obes. Surg..

[187] Lee P.C., Dixon J. (2017). Bariatric-metabolic surgery: A guide for the primary care physician. Aust. Fam. Physician.

[188] Sjöström L., Lindroos A.-K., Peltonen M., Torgerson J., Bouchard C., Carlsson B., Dahlgren S., Larsson B., Narbro K., Sjöström C.D. (2004). Lifestyle, Diabetes, and Cardiovascular Risk Factors 10 Years after Bariatric Surgery. N. Engl. J. Med..

[189] Loos R.J.F., Yeo G.S.H. (2022). The genetics of obesity: From discovery to biology. Nat. Rev. Genet..

[190] Di Lorenzo N., Antoniou S.A., Batterham R.L., Busetto L., Godoroja D., Iossa A., Carrano F.M., Agresta F., Alarçon I., Azran C. (2020). Clinical practice guidelines of the European Association for Endoscopic Surgery (EAES) on bariatric surgery: Update 2020 endorsed by IFSO-EC, EASO and ESPCOP. Surg. Endosc..

[191] Hedbäck N., Hindsø M., Bojsen-Møller K.N., Linddal A.K., Jørgensen N.B., Dirksen C., Møller A., Kristiansen V.B., Hartmann B., Holst J.J. (2022). Effect of Meal Texture on Postprandial Glucose Excursions and Gut Hormones After Roux-en-Y Gastric Bypass and Sleeve Gastrectomy. Front. Nutr..

[192] Tedjo D.I., Wilbrink J.A., Boekhorst J., Timmerman H.M., Nienhuijs S.W., Stronkhorst A., Savelkoul P.H.M., Masclee A.A.M., Penders J., Jonkers D.M.A.E. (2023). Impact of Sleeve Gastrectomy on Fecal Microbiota in Individuals with Morbid Obesity. Microorganisms.

[193] Browning M.G., Pessoa B.M., Khoraki J., Campos G.M. (2019). Changes in Bile Acid Metabolism, Transport, and Signaling as Central Drivers for Metabolic Improvements After Bariatric Surgery. Curr. Obes. Rep..

[194] Meek C.L., Lewis H.B., Reimann F., Gribble F.M., Park A.J. (2016). The effect of bariatric surgery on gastrointestinal and pancreatic peptide hormones. Peptides.

[195] Feris F., McRae A., Kellogg T.A., McKenzie T., Ghanem O., Acosta A. (2023). Mucosal and hormonal adaptations after Roux-en-Y gastric bypass. Surg. Obes. Relat. Dis..

[196] Peterli R., Steinert R.E., Woelnerhanssen B., Peters T., Christoffel-Courtin C., Gass M., Kern B., von Fluee M., Beglinger C. (2012). Metabolic and Hormonal Changes After Laparoscopic Roux-en-Y Gastric Bypass and Sleeve Gastrectomy: A Randomized, Prospective Trial. Obes. Surg..

[197] Cazzo E., Gestic M.A., Utrini M.P., Chaim F.D., Geloneze B., Pareja J.C., Chaim E.A., Magro D.O. (2016). GLP-2: A poorly understood mediator enrolled in various bariatric/metabolic surgery-related pathophysiologic mechanisms. Arq. Bras. Cir. Dig..

[198] Pournaras D.J., le Roux C.W. (2009). Obesity, gut hormones, and bariatric surgery. World J. Surg..

[199] Gao Z., Yang J., Liang Y., Yang S., Zhang T., Gong Z., Li M. (2022). Changes in Gastric Inhibitory Polypeptide (GIP) After Roux-en-Y Gastric Bypass in Obese Patients: A Meta-analysis. Obes. Surg..

[200] Quercia I., Dutia R., Kotler D., Belsley S., Laferrère B. (2014). Gastrointestinal changes after bariatric surgery. Diabetes Metab..

[201] Šebunova N., Štšepetova J., Kullisaar T., Suija K., Rätsep A., Junkin I., Soeorg H., Lember M., Sillakivi T., Mändar R. (2022). Changes in adipokine levels and metabolic profiles following bariatric surgery. BMC Endocr. Disord..

[202] Woelnerhanssen B., Peterli R., Steinert R.E., Peters T., Borbély Y., Beglinger C. (2011). Effects of postbariatric surgery weight loss on adipokines and metabolic parameters: Comparison of laparoscopic Roux-en-Y gastric bypass and laparoscopic sleeve gastrectomy—A prospective randomized trial. Surg. Obes. Relat. Dis..

[203] Svane M.S., Bojsen-Møller K.N., Martinussen C., Dirksen C., Madsen J.L., Reitelseder S., Holm L., Rehfeld J.F., Kristiansen V.B., van Hall G. (2019). Postprandial Nutrient Handling and Gastrointestinal Hormone Secretion After Roux-en-Y Gastric Bypass vs Sleeve Gastrectomy. Gastroenterology.

[204] Fatima F., Hjelmesæth J., Birkeland K.I., Gulseth H.L., Hertel J.K., Svanevik M., Sandbu R., Småstuen M.C., Hartmann B., Holst J.J. (2021). Gastrointestinal Hormones and β-Cell Function After Gastric Bypass and Sleeve Gastrectomy: A Randomized Controlled Trial (Oseberg). J. Clin. Endocrinol. Metab..

[205] Nannipieri M., Baldi S., Mari A., Colligiani D., Guarino D., Camastra S., Barsotti E., Berta R., Moriconi D., Bellini R. (2013). Roux-en-Y Gastric Bypass and Sleeve Gastrectomy: Mechanisms of Diabetes Remission and Role of Gut Hormones. J. Clin. Endocrinol. Metab..

[206] Berthoud H.-R., Shin A.C., Zheng H. (2011). Obesity surgery and gut–brain communication. Physiol. Behav..

[207] Lampropoulos C., Alexandrides T., Tsochatzis S., Kehagias D., Kehagias I. (2021). Are the Changes in Gastrointestinal Hormone Secretion Necessary for the Success of Bariatric Surgery? A Critical Review of the Literature. Obes. Surg..

[208] Svane M.S., Jørgensen N.B., Bojsen-Møller K.N., Dirksen C., Nielsen S., Kristiansen V.B., Toräng S., Albrechtsen N.J.W., Rehfeld J.F., Hartmann B. (2016). Peptide YY and glucagon-like peptide-1 contribute to decreased food intake after Roux-en-Y gastric bypass surgery. Int. J. Obes..

[209] Capristo E., Panunzi S., De Gaetano A., Spuntarelli V., Bellantone R., Giustacchini P., Birkenfeld A.L., Amiel S., Bornstein S.R., Raffaelli M. (2018). Incidence of Hypoglycemia After Gastric Bypass vs Sleeve Gastrectomy: A Randomized Trial. J. Clin. Endocrinol. Metab..

[210] Papamargaritis D., Koukoulis G., Sioka E., Zachari E., Bargiota A., Zacharoulis D., Tzovaras G. (2012). Dumping Symptoms and Incidence of Hypoglycaemia After Provocation Test at 6 and 12 Months After Laparoscopic Sleeve Gastrectomy. Obes. Surg..

[211] Masclee G.M.C., Masclee A.A. (2023). Dumping Syndrome: Pragmatic Treatment Options and Experimental Approaches for Improving Clinical Outcomes. Clin. Exp. Gastroenterol..

[212] Lupoli R., Lembo E., Ciciola P., Schiavo L., Pilone V., Capaldo B. (2020). Continuous glucose monitoring in subjects undergoing bariatric surgery: Diurnal and nocturnal glycemic patterns. Nutr. Metab. Cardiovasc. Dis..

[213] Hasan M.A., Schwartz S., McKenna V., Ing R. (2023). An Imbalance of Pathophysiologic Factors in Late Postprandial Hypoglycemia Post Bariatric Surgery: A Narrative Review. Obes. Surg..

[214] Salehi M., Vella A., McLaughlin T., Patti M.-E. (2018). Hypoglycemia After Gastric Bypass Surgery: Current Concepts and Controversies. J. Clin. Endocrinol. Metab..

[215] Jalleh R.J., Umapathysivam M.M., Plummer M.P., Deane A., Jones K.L., Horowitz M. (2023). Postprandial plasma GLP-1 levels are elevated in individuals with postprandial hypoglycaemia following Roux-en-Y gastric bypass—A systematic review. Rev. Endocr. Metab. Disord..

[216] Goldfine A.B., Mun E.C., Devine E., Bernier R., Baz-Hecht M., Jones D.B., Schneider B.E., Holst J.J., Patti M.E. (2007). Patients with Neuroglycopenia after Gastric Bypass Surgery Have Exaggerated Incretin and Insulin Secretory Responses to a Mixed Meal. J. Clin. Endocrinol. Metab..

[217] Salehi M., Gastaldelli A., D’Alessio D.A. (2014). Altered islet function and insulin clearance cause hyperinsulinemia in gastric bypass patients with symptoms of postprandial hypoglycemia. J. Clin. Endocrinol. Metab..

[218] Llewellyn D.C., Ellis H.L., Aylwin S.J.B., Oštarijaš E., Green S., Sheridan W., Chew N.W.S., le Roux C.W., Miras A.D., Patel A.G. (2023). The efficacy of GLP-1RAs for the management of postprandial hypoglycemia following bariatric surgery: A systematic review. Obesity.

[219] Elhag W., Lock M., El Ansari W. (2023). When Definitions Differ, are Comparisons Meaningful? Definitions of Weight Regain After Bariatric Surgery and Their Associations with Patient Characteristics and Clinical Outcomes—A Need for a Revisit?. Obes. Surg..

[220] Sumithran P., Prendergast L.A., Delbridge E., Purcell K., Shulkes A., Kriketos A., Proietto J. (2011). Long-Term Persistence of Hormonal Adaptations to Weight Loss. N. Engl. J. Med..

[221] El Ansari W., Elhag W. (2021). Weight Regain and Insufficient Weight Loss After Bariatric Surgery: Definitions, Prevalence, Mechanisms, Predictors, Prevention and Management Strategies, and Knowledge Gaps—A Scoping Review. Obes. Surg..

[222] Martins C. (2021). Do we really know what drives relapse in obesity management?. Eur. J. Intern. Med..

[223] Vaccaro S., Itani L., Scazzina F., Bonilauri S., Cartelli C.M., El Ghoch M., Pellegrini M. (2022). Do Lifestyle Interventions before Gastric Bypass Prevent Weight Regain after Surgery? A Five-Year Longitudinal Study. Nutrients.

[224] Poitou C., Puder L., Dubern B., Krabusch P., Genser L., Wiegand S., Verkindt H., Köhn A., von Schwartzenberg R.J., Flück C. (2021). Long-term outcomes of bariatric surgery in patients with bi-allelic mutations in the POMC, LEPR, and MC4R genes. Surg. Obes. Relat. Dis..

[225] Cooiman M., Kleinendorst L., Aarts E., Janssen I., van Amstel H.P., Blakemore A., Hazebroek E., Meijers-Heijboer H., van der Zwaag B., Berends F. (2020). Genetic Obesity and Bariatric Surgery Outcome in 1014 Patients with Morbid Obesity. Obes. Surg..

[226] Campos A., Cifuentes L., Hashem A., Busebee B., Hurtado-Andrade M.D., Ricardo-Silgado M.L., McRae A., De la Rosa A., Feris F., Bublitz J.T. (2022). Effects of Heterozygous Variants in the Leptin-Melanocortin Pathway on Roux-en-Y Gastric Bypass Outcomes: A 15-Year Case–Control Study. Obes. Surg..

[227] Torrego-Ellacuría M., Barabash A., Matía-Martín P., Sánchez-Pernaute A., Torres A.J., Calle-Pascual A.L., Rubio-Herrera M.A. (2023). Combined Effect of Genetic Variants on Long-Term Weight Response after Bariatric Surgery. J. Clin. Med..

[228] Goni L., Cuervo M., Milagro F.I., Martínez J.A. (2015). A genetic risk tool for obesity predisposition assessment and personalized nutrition implementation based on macronutrient intake. Genes Nutr..

[229] Bonetti G., Dhuli K., Ceccarini M.R., Kaftalli J., Samaja M., Precone V., Cecchin S., Maltese P.E., Guerri G., Marceddu G. (2022). Next-Generation Sequencing of a Large Gene Panel for Outcome Prediction of Bariatric Surgery in Patients with Severe Obesity. J. Clin. Med..

[230] van der Meer R., Mohamed S.A., Monpellier V.M., Liem R.S.L., Hazebroek E.J., Franks P.W., Frayling T.M., Janssen I.M.C., Serlie M.J. (2023). Genetic variants associated with weight loss and metabolic outcomes after bariatric surgery: A systematic review. Obes. Rev..

[231] Faccioli N., Poitou C., Clément K., Dubern B. (2023). Current Treatments for Patients with Genetic Obesity. J. Clin. Res. Pediatr. Endocrinol..

[232] Updated June, 2021. www.who.int/newsroom/fact-sheets/detail/obesity-and-overweight.

[233] (2024). American Diabetes Association Professional Practice Committee. Obesity and Weight Management for the Prevention and Treatment of Type 2 Diabetes: Standards of Care in Diabetes–2024. Diabetes Care.

[234] Mingrone G., Panunzi S., De Gaetano A., Guidone C., Iaconelli A., Nanni G., Castagneto M., Bornstein S., Rubino F. (2015). Bariatric–metabolic surgery versus conventional medical treatment in obese patients with type 2 diabetes: 5 year follow-up of an open-label, single-centre, randomised controlled trial. Lancet.

[235] Yska J.P., van Roon E.N., de Boer A., Leufkens H.G.M., Wilffert B., de Heide L.J.M., de Vries F., Lalmohamed A. (2015). Remission of Type 2 Diabetes Mellitus in Patients After Different Types of Bariatric Surgery. JAMA Surg..

[236] Jackson H.T., Anekwe C., Chang J., Haskins I.N., Stanford F.C. (2019). The Role of Bariatric Surgery on Diabetes and Diabetic Care Compliance. Curr. Diabetes Rep..

[237] Svane M.S., Bojsen-Møller K.N., Nielsen S., Jørgensen N.B., Dirksen C., Bendtsen F., Kristiansen V.B., Hartmann B., Holst J.J., Madsbad S. (2016). Effects of endogenous GLP-1 and GIP on glucose tolerance after Roux-en-Y gastric bypass surgery. Am. J. Physiol. Metab..

[238] Ferrannini E., Mingrone G. (2009). Impact of Different Bariatric Surgical Procedures on Insulin Action and β-Cell Function in Type 2 Diabetes. Diabetes Care.

[239] Sandoval D.A., Patti M.E. (2023). Glucose metabolism after bariatric surgery: Implications for T2DM remission and hypoglycaemia. Nat. Rev. Endocrinol..

[240] Kim K.S., Sandoval D.A. (2017). Endocrine Function after Bariatric Surgery. Compr. Physiol..

[241] Mosinski J.D., Aminian A., Axelrod C.L., Batayyah E., Romero-Talamas H., Daigle C., Mulya A., Scelsi A., Schauer P.R., Brethauer S.A. (2021). Roux-en-Y gastric bypass restores islet function and morphology independent of body weight in ZDF rats. Am. J. Physiol. Metab..

[242] Abu-Gazala S., Horwitz E., Schyr R.B.-H., Bardugo A., Israeli H., Hija A., Schug J., Shin S., Dor Y., Kaestner K.H. (2018). Sleeve Gastrectomy Improves Glycemia Independent of Weight Loss by Restoring Hepatic Insulin Sensitivity. Diabetes.

[243] Kirwan J.P., Münzberg H., Berthoud H.-R. (2018). Mechanisms Responsible for Metabolic Improvements of Bariatric Surgeries. Diabetes.

[244] Scheen A.J. (2023). Use of SGLT2 inhibitors after bariatric/metabolic surgery: Risk/benefit balance. Diabetes Metab..

[245] Alabduljabbar K., le Roux C.W. (2023). Pharmacotherapy before and after bariatric surgery. Metabolism.

[246] Wachsmuth H.R., Weninger S.N., Duca F.A. (2022). Role of the gut–brain axis in energy and glucose metabolism. Exp. Mol. Med..

[247] Frias J.P., Deenadayalan S., Erichsen L., Knop F.K., Lingvay I., Macura S., Mathieu C., Pedersen S.D., Davies M. (2023). Efficacy and safety of co-administered once-weekly cagrilintide 2·4 mg with once-weekly semaglutide 2·4 mg in type 2 diabetes: A multicentre, randomised, double-blind, active-controlled, phase 2 trial. Lancet.

